# Influence of Malted Chickpea on the Composition of Volatiles in Hummus

**DOI:** 10.3390/molecules30061231

**Published:** 2025-03-10

**Authors:** Alan Gasiński, Luis Noguera-Artiaga, Joanna Kawa-Rygielska

**Affiliations:** 1 Department of Fermentation and Cereals Technology, Wrocław University of Environmental and Life Sciences, Chełmońskiego 37, 51-630 Wrocław, Poland; joanna.kawa-rygielska@upwr.edu.pl; 2 Grupo de Investigación en Calidad y Seguridad Alimentaria, Instituto de Investigación e Innovación Agroalimentaria y Agroambiental (CIAGRO-UMH), Universidad Miguel Hernández, Carretera de Beniel, km 3,2, 03312 Alicante, Spain; lnoguera@umh.es

**Keywords:** germination, drying, malting

## Abstract

In recent years, research has shown that malting legume seeds can be a viable modification method; however, very few applications of legume malts are currently available. This research aimed to determine whether using malted chickpeas can significantly impact the volatile composition of the produced hummus, as aroma is one of the crucial factors in the acceptance of food products. Five chickpea malts produced by germination by a different amount of time (24 h, 48 h, 72 h, 96 h, and 120 h) were used as a substrate for the production of hummuses and were compared to the hummus produced from unmalted chickpeas. Hummuses produced from the chickpea malt germinated for 96 h and 120 h were characterized by a higher concentration of most volatiles than the control sample, while the hummuses produced from chickpea malts germinated for 24 h, 48 h, and 72 h were characterized by a lower concentration of volatiles.

## 1. Introduction

Hummus is a dip or a spread produced from boiled chickpeas (*Cicer arietinum* L.). It is known for its creamy texture, flavor, and versatility, traditionally consisting of a balanced combination of chickpeas, tahini, lemon juice, garlic, and olive oil [1]. However, due to the diversification of global culinary preferences, there is growing interest in exploring novel ingredients and preparation techniques, even in traditional food products, such as hummus [2]. As chickpeas are the primary ingredient in hummus, any modifications applied to them are expected to significantly influence the aroma of the final product. One widely used technique for modifying plant seeds is malting, which is primarily applied to cereals [3]. Malting involves controlled hydration, germination, and drying of the seeds, which significantly changes the flavor and aroma of the product. Recent studies have demonstrated that malting can also be applied to various legume seeds, such as beans and lentils, impacting both their aroma and the composition of various antinutritional factors (such as the content of phytic acid or raffinose family oligosaccharides) [4,5]. During malting, various substances are released in the germinating seed, such as sugars or amino acids, which can then undergo so-called Maillard reactions during the drying stage of the malting and result in an increase of various flavor-active components present in the malted seed [6]. Additionally, during germination, certain enzymes, such as lipoxygenases, can transform various components of the seed into volatile compounds of diverse tastes and odor. One of the crucial reactions of these types is a chain of reactions that changes triglycerides into flavor-active aldehydes present in the malts [7]. Previous research about malting legumes (lentil seeds) has shown that malted legumes contain significantly different concentrations of volatiles than non-modified seeds [8]. However, the information about the use of legume seed malts in food production is actually very limited. Studies mainly concentrate on the technological properties of these malts. Current research about the uses of legume malts is mainly restricted to the use of legume malts in the brewing industry, which is not a typical technology where legume seeds are generally used.

Additionally, analysis of the volatile composition of the fermented products concentrates mainly on the components produced through the process of fermentation, not on the volatiles introduced from the malt [9]. It seems that the interesting avenue of research suitable for the analysis of legume malts would be the branches of the food industry, which typically rely on the use of legume seeds, not on malts produced from grains. As the legume malts are significantly different in many aspects from the unmalted legume seeds, the investigation of the use of these malts in various branches of the food industry can be an interesting and promising avenue for future research. The choice of ingredients in hummus production is pivotal in determining its sensory attributes, particularly its aroma. Aroma, an integral component of flavor perception, relies on a complex interplay of volatile organic compounds (VOCs) that emanate from the food matrix [2,10,11]. The volatile profile of hummus is influenced by several factors, including chickpea variety, processing methods, and ingredient proportions [12,13,14]. As mentioned before, malting significantly changes the odor of the seeds; therefore, malts produced from the chickpeas used as a hummus ingredient could contribute to unique product flavor [6,7]. The goal of this study is to determine whether various chickpea malts (germinated by different lengths of time) and various contributions of the chickpea malt during hummus production can significantly change the aroma of the finished product.

## 2. Results and Discussion

The research consisted of an analysis of volatile compounds in six different hummuses, five produced from malted chickpeas and one produced from unmalted chickpeas (0D). The hummuses produced from malted chickpeas were differentiated by various lengths of germination. Sample 1D was produced from malt germinated for 24 h, sample 2D was produced from malt germinated for 48 h, sample 3D was produced from malt germinated for 72 h, sample 4D was produced from malt germinated for 96 h, and sample 5D was produced from malt germinated for 120 h. Gas chromatography coupled with mass spectrometry (GC-MS) allowed for the identification and relative quantification (using the internal standard method) of 22 volatile compounds in the hummus samples produced from malted chickpeas, germinated by various amounts of time (24–120 h). Table 1 shows the Kovats indices of the identified components, the percentage of the similarity search from the NIST library, as well as the perceived odor of the identified compounds and odor threshold. Table 2 shows the concentration of volatiles in the hummus samples measured using GC-MS utilizing internal standards for quantitation. Sample chromatograms and mass spectrum search results were provided in Supplementary Data.

**Figure 1:**
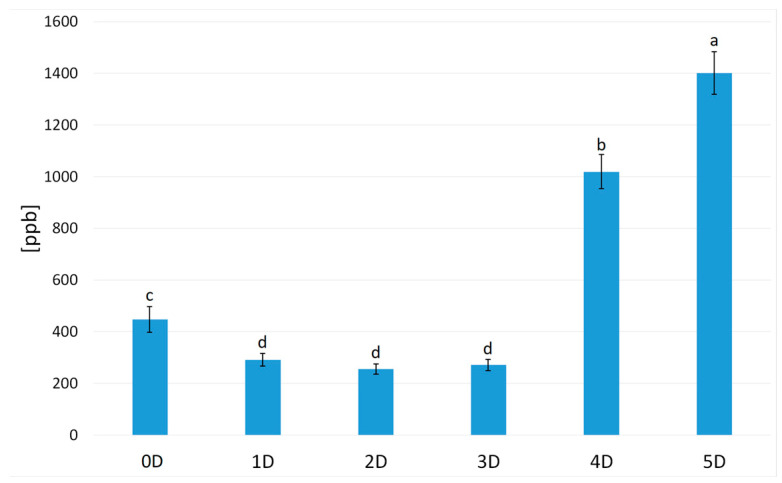
The total relative concentration of volatiles in the hummus samples. Letters (a, b, c, d) indicate homogenous groups (Tukey test, α = 0.05).

Most of the detected compounds belonged to the chemical group of aldehydes (nine compounds) and alcohols (five compounds). In the volatilome of hummus, three ketones, three pyrazines, one furan, and one terpene were also detected. The total concentration of volatiles in sample 0D was 448.03 ppb (Figure 1). Malts produced from chickpeas germinated for 24, 48, and 72 h were characterized by a lower concentration of volatiles, 291.15 ppb for 1D, 255.98 ppb for 2D, and 271.68 ppb for 3D. Germinating chickpeas by 96 or 120 h increased the total concentration of volatiles (1018.39 for 4D and 1401.67 ppb for 5D).

1-Hexanol is an alcohol associated with a beany, grassy off-flavor. However, its concentration in 1D, 2D, and 3D was approximately half that of the two times or almost two times smaller than in the 0D sample. This suggests that malts germinated for a short period of time (24–72 h) could be used in the production of hummus with a less intensive legume odor [29]. Ketones in legumes and malts are typically produced by lipoxygenases through the breakdown of fatty acid hydroperoxides [30]. One such ketone, 2-heptanone, is characterized by a fruity, spicy odor [31]. Samples 0D, 1D, 2D, and 3D contained similar amounts of this compound, while its concentration increased significantly in 4D and 5D. This may indicate that lipoxygenase activity in chickpea seeds increases substantially only after 72 h of germination. Heptanal, an aldehyde formed via the autooxidation of oleic and linoleic acid—both present in the components of hummus—was found in the highest concentrations in 4D and 5D [32,33].

In contrast, 1D, 2D, and 3D contained over two times less heptanal than 0D. These results suggest that chickpea seeds inherently contain small amounts of this aldehyde, which may be reduced during the malting process, particularly through steeping or drying. However, by 96–120 h of germination, conditions appear favorable for the subsequent autooxidation of the fatty acids, leading to increased heptanal formation [32,33]. 2,5-Dimethylpyrazine is typically formed through the Maillard reaction between dipeptides and reducing sugars [34]. 4D and 5D contained significantly greater amounts of this compound than other samples, which exhibited similar 2,5-dimethylpyrazine levels. This suggests that the activity of hydrolytic enzymes, capable of releasing substrates for the Maillard reaction in which 2,5-dimethylpyrazine is formed, may become more active after 96 h or 120 h of germination.

Unfortunately, the gathered data do not allow for pinpointing the activity of one particular enzyme or group of enzymes responsible for limiting the formation of the 2,5-dimethylpyrazine in 0D, 1D, 2D, and 3D. Future studies focusing on this aspect could provide valuable insights into the enzymatic processes influencing the volatile profile of malted chickpea hummus. Benzaldehyde is another compound that can be formed through the Maillard reaction [32,33]. Its primary precursor is the amino acid phenylalanine [35]. Characterized by an almond-like odor, benzaldehyde contributes to the aroma of various food products [36]. The germination time of the chickpea malts had a significant effect on the concentration of benzaldehyde. During the first three days of germination, benzaldehyde levels decreased, reaching their lowest concentration in the 3D sample. However, a significant increase was observed in samples 4D and 5D, where benzaldehyde content more than doubled compared to 0D. It is possible that benzaldehyde functions as a growth mediator and is only released after a few days of germination, but the elevated benzaldehyde level after four and five days of germination could also be the result of a higher concentration of phenylalanine and reducing sugars in germinating chickpea seeds [37]. The concentration of heptanol followed a similar pattern, decreasing in 1D, 2D, and 3D before increasing two- to three-fold in 4D and 5D. A study by Mao et al. (2024) [38] demonstrated that heptanol levels could either decrease or increase during germination, though the effect was variety-specific and was not measured across different germination durations.

Another volatile compound detected in hummus was 1-octen-3-ol, an alcohol characterized by undesirable ‘beany’ off-flavor; however, it is detected at a concentration of at least 100 ppb. Hummuses from the malted chickpeas contained a maximal 34.09 ppb of this alcohol, while 0D contained 12.37 ppb; therefore, changes to the level of this component due to the use of malted chickpeas in the hummus production should not deteriorate the aroma of the finished product [39]. The ketone 6-metyl-5-hepten-2-one, known for its fruity odor, has been previously detected in various legume seeds [40,41,42]. However, there is currently no consensus on whether the germination process increases or decreases its concentration [40,43]. In the analyzed hummuses, 4D and 5D contained the highest levels of this compound, followed by 0D, while 1D, 2D, and 3D exhibited the lowest concentrations. The concentration of 2-pentylfuran in 5D was five times greater than in 0D, whereas 1D, 2D, and 3D contained lower levels of this compound. This again suggests that chickpea malts germinated for 24–72 h exhibit significantly lower enzymatic activity than chickpea malts germinated for 96–120 h. Since 2-pentylfuran, which contributes a beany flavor, is primarily formed from linoleic acid lipoxygenase activity, its increased presence in longer germinated samples supports this hypothesis [44]. The concentration of 2-ethyl-3-methyl pyrazine followed a pattern similar to that of 2,5-dimethylpyrazine, with the highest levels observed in 4D and 5D. However, unlike 2,5-dimethylpyrazine, the concentration of 2-ethyl-3-methylpyrazine in 1D was lower than in 0D. The discrepancy may be difficult to explain, as 2-ethyl-3-methylpyrazine, unlike 2,5-dimethypyrazine, is a key aroma-active compound found in tahini paste [45]. Octanal, which has a ‘soapy’ and ‘fatty’ aroma [46], exhibited similar concentrations in 0D and 1D but decreased significantly in 2D.

In contrast, its concentration in 4D was three times higher than in 0D, while in 5D, it was over five times higher. Research by Rajhi et al. (2022) [43] has shown that germination generally increases octanal concentration in legume seeds. The concentration of 2-ethyl-1-hexanol remained similar across most samples, except for 1D, which had the lowest concentration. This alcohol, characterized by a rose-like aroma, is typically produced in the later stages of the chickpea development as a phytochemical with antifungal properties against *Fusarium* [47,48]. 3-Octen-2-one, another compound produced through oxidation of polyunsaturated fatty acids, was most abundant in 5D, where its concentration was twice as high as in 0D [39]. Similarly, 1D, 2D, and 3D contained lower levels of this compound compared to 0D. A comparable trend was observed in benzeneacetaldehyde, with the highest concentrations detected in 4D and 5D and the lowest detected in 1D, 2D, and 3D. Benzeneacetaldehyde can be formed via the Strecker degradation pathway, Maillard reactions, or the oxidation of free fatty acids. Due to the complexity of these pathways, it is difficult to determine which precursor was in the lower concentration in the hummus samples [49,50,51]. However, these findings strongly suggest that 1D, 2D, and 3D exhibited lower enzymatic activity compared to the other samples. β-Ocimene was the only terpene detected in the prepared hummuses. The highest concentration was found in 0D, while all hummus samples prepared from malted chickpeas contained lower amounts. This terpene is naturally present in the legume seeds and may function as an insect repellent, which could explain its higher concentration in the hummus prepared from the unmalted chickpea [52,53]. 1-Octanol, an alcohol with an oily, aldehydic odor, can be generated through lipoxygenase enzyme activity [39,54]. As observed with many other compounds lipoxygenase-derived compounds, its highest concentrations were detected in 4D and 5D, while the lowest levels were found in 1D, 2D, and 3D. 3-Ethyl-2,5-dimethyl-pyrazine is characterized by a pleasant, nutty odor and has previously been identified in thermally treated chickpea products [55]. This pyrazine is typically formed through thermal amino acid generation, with 4D and 5D exhibiting the highest concentrations [56]. The lowest concentration was found in 2D. This suggests that chickpeas germinated for 96 h or 120 h contain the highest levels of free amino acids. However, it remains unclear whether this results from increased proteolytic activity or simply from the accumulation of amino acids due to extended germination.

Further research is needed to confirm these findings, but producing hummus from chickpea malts germinated for 96 h or 120 h could enhance the ‘nutty’ aroma of the product, as this compound is perceptible at very low concentrations [20]. Nonanal, an aldehyde primarily formed via linoleic acid oxidation, exhibited the highest concentration in 5D, nearly six times greater than in 0D [57,58]. Interestingly, nonanal was one of the few volatiles with significantly higher levels in 5D compared to 4D. This suggests that 5D may have either higher LOX activity or increased lipase activity, leading to a greater release of linoleic acid. Alternatively, the accumulation of free fatty acids in 5D might have facilitated higher nonanal formation during the drying process. However, to confirm these hypotheses, further studies on LOX and lipase activity in chickpeas would be necessary. Unlike nonanal, trans-2-nonenal, a key off-flavor compound in barley malts, did not exhibit drastic differences in concentration among the samples [7,59]. Multiple formation pathways have been proposed for trans-2-nonenal, including fatty acid oxidation, Maillard reaction, Strecker degradation of amino acids, oxidation of higher alcohols, and secondary oxidation of long-chain aldehydes [7]. In the analyzed hummus samples, trans-2-nonenal was present at relatively low levels, suggesting that its formation in hummus may differ from the typical lipoxygenase-driven pathway seen in the formation of other aldehydes. However, as with other volatiles, 4D and 5D contained the highest amounts of this compound, while 0D exhibited a concentration of this aldehyde compared to 2D and 3D. Trans-2-nonenal was not detected in 1D. Decanal, another aldehyde, showed considerable variation among the samples. Its concentration in 5D was nearly ten times higher than in 0D, while in 4D, it was slightly over three times higher. Interestingly, 1D and 3D also exhibited higher decanal levels than 0D, whereas 2D contained less than 0D. These results indicate that the decanal concentration is highly influenced by the germination period. Although the most probable source of decanal in legume seeds is fatty acid oxidation, the precise mechanism remains unclear, making it difficult to fully explain the observed changes in its concentration in hummus from malted chickpeas [37,60].

Undecanal was the only volatile compound, which was not present in the 0D but present in small amounts in all hummus samples produced from the malted chickpeas. This suggests that undecanal formation may primarily result from thermally induced lipid oxidation occurring in the malt during the drying [61]. Similarly, dodecanal, an aldehyde typically found in legumes in trace amounts, can originate from the oxidative degradation of palmitic acid [62]. However, the results of this study indicate that the process of malting does not significantly increase dodecanal levels in hummus. These findings demonstrate that using malted chickpeas in hummus production can alter its volatile profile, potentially influencing its aroma. The duration of chickpea germination could be manipulated to enhance or reduce the concentrations of specific aroma-active components in the final product. Future studies exploring consumer preferences for hummus with varying volatile profiles could provide valuable insights into the sensory appeal of these products across different populations.

## 3. Materials and Methods

### 3.1. Materials

#### 3.1.1. Raw Material

The raw materials used in this study were chickpea seeds (*Cicer arietinum* L.) of the Kabuli variety, olive oil (Monini Classico, produced by Monini Company, Spoleto, Italy), and tahini paste (100% sesame seeds, produced by Naturalnie Zdrowe, Wiązowana Polska, Poland).

#### 3.1.2. Reagents and Standards

Standards used in this study were: 1-hexanol (99%), 2-heptanone (99%), 2,5-dimethylpyrazine (98%), benzaldehyde (99%), 1-heptanol (99%), 1-octen-3-ol (99%), 2-pentylfuran (98%), 2-ethyl-3-methylpyrazine (98%), octanal (99%), 2-ethyl-1-hexanol (99%), 3-octen-2-one (99%), benzeneacetaldehyde, β-ocimene (90%, mix of isomers), 1-octanol (99%), nonanal (99%), trans-2-nonenal (99%), decanal (99%), undecanal (99%), 2-undecanone (99%), and dodecanal (99%). All standards were acquired from Merck KGaA (Darmstadt, Germany). Citric acid and sodium chloride (Chempur, Piekary Śląskie, Poland) were used as an additive to the produced hummus. The internal standard used in the GC-MS analysis was 2-undecanone in cyclohexane solution (1 mg/1 dm^3^).

### 3.2. Methods

#### 3.2.1. Chickpea Malt Production

**Figure 2:**
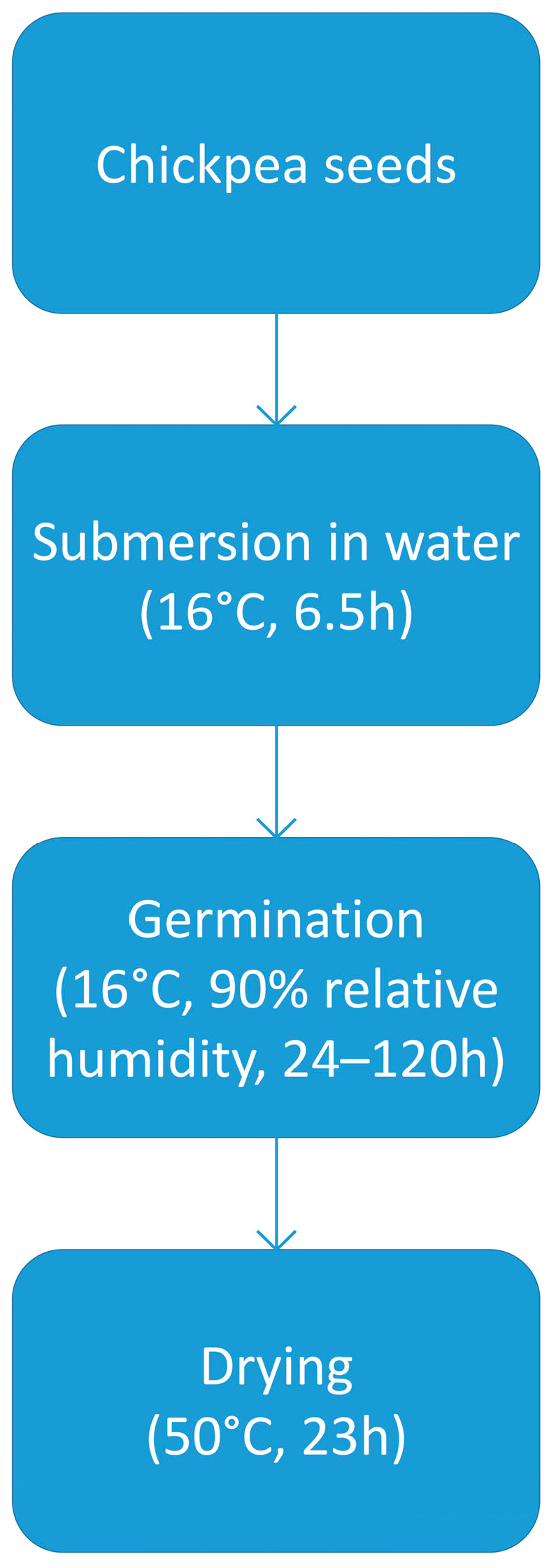
Simplified diagram of the chickpea malting process.

Sixty grams of chickpea seeds with a known moisture content of 10 ± 1% (measured using Brabender MT moisture analyzer, Brabender GmbH & Co, Duisburg, Germany) were transferred to a stainless steel perforated container. The simplified malt production process is presented in Figure 2.

Fifteen containers filled with chickpeas were submerged in water at 16 °C for 6.5 h. After soaking, the containers were removed from the water, weighed to assess the moisture content (52–54%), and transferred to the KK240 Smart-Pro germination chamber (Pol-Eko Aparatura, Wodzisław Śląski, Poland) set at 16 °C and 90% relative humidity. During germination, the containers were weighed daily, and chickpea seeds were sprinkled with sterile, distilled water and mixed. At 24 h intervals (24 h, 48 h, 72 h, 96 h, and 120 h), three containers were removed from the germination chamber and transferred to UF110 Plus Dryer (Memmert GmbH + Co, Schwabach, Germany) where they were dried at 50 °C for 23 h. This process resulted in the production of five distinct chickpea malts, germinated for 24 h, 48 h, 72 h, 96 h, and 120 h.

#### 3.2.2. Hummus Production

Chickpea seeds or chickpea malt (45 g) were soaked in 180 cm^3^ of water (20 °C) for 16 h. After soaking, the seeds/malts were strained, rinsed with cold water, and transferred to a beaker containing 112.5 cm^3^ of boiling water. They were boiled for 30 min, then strained, rinsed, and cooled. Boiled chickpea seeds/malts were then blended with 15 g of tahini paste, 4.5 g of olive oil, 0.5 g of NaCl, and 0.5 g of citric acid using a kitchen blender. This process resulted in six different hummus types:

- Hummus produced from unmalted chickpea seeds (0D);
- Hummus produced from chickpea malt germinated for 24 h (1D);
- Hummus produced from chickpea malt germinated for 48 h (2D);
- Hummus produced from chickpea malt germinated for 72 h (3D);
- Hummus produced from chickpea malt germinated for 96 h (4D);
- Hummus produced from chickpea malt germinated for 120 h (5D).

Each type of hummus was prepared in triplicate.

#### 3.2.3. Extraction and Analysis of Volatile Compounds in the Hummus

The analysis of volatile compounds in hummus was performed using the HS-SPME-GC-MS method. One gram of the hummus sample was transferred to the 20 cm^3^ headpace vial. Five cm^3^ of saturated sodium chloride water solution (40 °C) and 50 ng of internal standard (2-undecanone in cyclohexane, 1 mg/1 dm^3^) were added to the vial along with a stir bar. The vial was sealed with a screw cap with a septum and placed on a magnetic stirrer heatplate set at 40 °C and 200 rpm. The SPME holder needle was then inserted into the vial through the septum. After 5 min of temperature equilibration, SPME fiber (1 cm DVB/CAR/PDMS, 50/30 μm, Supelco, Bellefonte, PA, USA) was exposed to the headspace of the sample for 20 min. After extraction, the fiber was retracted and inserted into the gas chromatograph injection port for desorption. The desorption time in the injection port was 2 min. Gas chromatography and mass spectrometry of the volatiles were conducted using a GC-2010 Plus chromatograph coupled with GCMS-QP2010 SE mass spectrometer (Shimadzu, Kyoto, Japan), equipped with ZB-5 column (Phenomenex, Torrance, CA, USA) (30 m length × 0.25 mm internal diameter × 0.25 μm film thickness). The injection port temperature was maintained at 195 °C. Analyses were carried out using helium as a carrier gas at a flow rate of 1.78 cm^3^/min and a starting pressure of 100 kPa. The oven temperature program was as follows: initial temperature of 40 °C (held for 1 min); ramp up at 8 °C/min to 195 °C, and final hold at 195 °C for 5 min. The ion source temperature was set at 250 °C, while the interface temperature was maintained at 195 °C. Scanning was performed in the 35–350 m/z range using 70 mV electron ionization, with an event time of 0.3 s (scan speed:1111). Absolute quantification was not performed, and results are based on relative quantification via the internal standard. Each hummus sample was analyzed in duplicate (a total of six analyses for each hummus type).

#### 3.2.4. Data Analysis

The volatile compounds were identified by comparing retention indices to Kovats standards and NIST17 chemical standard libraries and comparing the retention time and mass spectra of the chemical standard. Quantification was performed using the internal standard method. Chromatographic peaks were integrated using the Shimadzu PostRun Analysis program (Shimadzu, Kyoto, Japan). Results of the analysis of the concentration of volatiles in hummus were statistically analyzed in the Statistica 13 program from Statsoft (Tulsa, OK, USA) using one-way ANOVA (α = 0.05). Tukey’s test was used to determine homogenous groups. Limits of detection and limits of quantitation are presented in Table 3.

## 4. Conclusions

Hummuses produced from malted chickpeas are characterized by a different amount of volatiles than hummus produced from unmalted chickpeas. However, the germination length of the chickpea during the malting procedure has a crucial influence on the amount of volatiles present in the finished product. The main groups of compounds that are influenced by the malting procedure are aldehydes, alcohols, ketones, and pyrazines. Hummuses produced from malts germinated by 96 h or 120 h were mostly characterized by a greater amount of each volatile compound than hummus produced from unmalted chickpeas, while using chickpeas germinated by 24 h, 48 h, and 72 h resulted in a lower concentration of most of the volatiles. It is possible, therefore, to choose various kinds of malted chickpeas in order to produce hummus with a stronger or milder odor. Furthermore, the gathered data show that legume malts, due to their modified volatile composition, could be an interesting topic of research in all branches of food science that use legume seeds and could provide more interesting information in the future.

## Figures and Tables

**Table 1:** Kovats indices, similarity search, perceived odor, and odor threshold of the identified volatile compounds.

Compound	KI Exp.	KI Lit. ^1^	Similarity Search	Perceived Odor of the Compound ^2^	Odor Threshold [ppb] ^3^
1-Hexanol	849	863	95%	green; herbaceous; woody; sweet, beany; grassy	2500
2-Heptanone	874	889	95%	fruity; spicy; sweet; herbal; coconut; woody	224
Heptanal	891	902	96%	fresh; aldehydic; fatty; green; herbal; cognac; ozone	2.8
Pyrazine, 2,5- dimethyl-	905	911	92%	nutty; peanutty; musty; earthy; powdery; roasted; cocoa-like.	7.9
Benzaldehyde	949	960	94%	almond; fruity; nutty.	350
1-Heptanol	962	966	92%	oily; nutty; fatty; green; aldehydic	520
1-Octen-3-ol	974	980	93%	beany, mushroom; fungal; earthy; floral.	100
5-Hepten-2-one, 6-methyl-	979	985	96%	fatty; green; citrusy.	525
Furan, 2-pentyl	984	988	95%	fruity; green; earthy; beany	4.8
Pyrazine, 2-ethyl-3-methyl-	999	1002	96%	nutty; peanut; musty; corn-like; earthy; bready.	0.55
Octanal	1004	998	93%	aldehydic; waxy; citrusy; soapy, fatty, orange-like.	3.4
1-Hexanol, 2-ethyl	1008	1002	91%	rosey; citrusy; fresh; floral; oily; sweet.	1493
3-Octen-2-one	1028	1030	94%	earthy; spicy; herbal; sweet; mushroom; hay-like; blueberry.	6.7
Benzeneacetaldehyde	1046	1042	92%	honey; sweet; floral; chocolate; cocoa	4
β-Ocimene	1052	1050	91%	floral; herbal; flowery; sweet.	34
1-Octanol	1061	1068	94%	waxy; green; citrusy; aldehydic; oily, floral; sweet; fatty; coconutty.	4.4
Pyrazine, 3-ethyl-2,5-dimethyl-	1079	1074	96%	potato-like; cocoa; roasted; nutty.	0.00186
Nonanal	1108	1100	94%	waxy; aldehydic; citrusy; fresh; green; lemon peel; cucumber-like; fatty.	2.8
*trans*-2-Nonenal	1159	1149	93%	beany; green; soapy; cucumber-like; melon; aldehydic; fatty.	0.19
Decanal	1213	1201	94%	sweet; aldehydic; orange; waxy; citrus rind.	9.3
Undecanal	1314	1306	92%	waxy; aldehydic; soapy; citrusy.	5
Dodecanal	1419	1408	91%	soapy; waxy; citrus; orange; mandarin.	0.53

^1^ Reference for the Kovats Index library: [15]; ^2^ Reference for the perceived odors: [16]; ^3^ References for odor threshold: [17,18,19,20,21,22,23,24,25,26,27,28].

**Table 2:** The relative concentration of volatile compounds [ppb] in hummus produced from malted and unmalted chickpeas was quantified using the internal standard method.

Compound	0D ^1^	1D	2D	3D	4D	5D
1-Hexanol	136.45 ± 21.32 c	61.37 ± 14.66 d	66.82 ± 6.48 d	71.55 ± 11.19 d	335.48 ± 23.59 b	434.21 ± 34.21 a
2-Heptanone	15.26 ± 4.57 b	18.20 ± 3.42 b	13.47 ± 3.26 b	18.07 ± 2.56 b	44.55 ± 19.07 a	44.32 ± 18.19 a
Heptanal	15.22 ± 2.78 b	7.60 ± 1.69 c	7.04 ± 1.67 c	7.50 ± 1.63 c	44.23 ± 13.78 a	53.02 ± 9.56 a
Pyrazine, 2,5- dimethyl-	15.03 ± 1.16 b	17.27 ± 1.97 b	16.24 ± 1.25 b	16.69 ± 1.27 b	23.18 ± 5.74 a	23.73 ± 4.21 a
Benzaldehyde	16.50 ± 3.26 bc	10.76 ± 3.33 cd	12.10 ± 1.39 c	9.55 ± 0.82 d	35.16 ± 7.44 a	29.85 ± 8.42 a
1-Heptanol	10.87 ± 1.02 c	16.16 ± 3.05 b	6.41 ± 0.58 d	4.70 ± 0.37 e	23.19 ± 3.76 a	22.22 ± 3.59 a
1-Octen-3-ol	12.37 ± 2.48 b	6.18 ± 0.75 d	7.32 ± 0.30 c	6.41 ± 0.66 cd	27.02 ± 9.86 a	34.09 ± 11.05 a
5-Hepten-2-one, 6-methyl-	17.41 ± 3.45 bc	11.88 ± 1.81 d	9.74 ± 1.25 d	8.99 ± 2.35 d	37.63 ± 15.11 ab	33.67 ± 10.81 ab
Furan, 2-pentyl	21.89 ± 8.21 c	8.96 ± 2.84 e	12.97 ± 2.50 d	14.96 ± 1.75 d	62.98 ± 23.02 b	114.80 ± 28.42 a
Pyrazine, 2-ethyl-3-methyl-	9.99 ± 1.37 b	6.69 ± 1.05 c	9.33 ± 0.67 b	7.33 ± 0.93 bc	19.66 ± 6.19 a	23.32 ± 5.34 a
Octanal	15.57 ± 4.09 cd	15.33 ± 6.43 cd	6.28 ± 2.30 e	11.32 ± 2.01 d	47.55 ± 11.63 b	82.34 ± 20.87 a
1-Hexanol, 2-ethyl	7.29 ± 0.33 a	5.27 ± 0.39 b	6.54 ± 1.11 ab	6.63 ± 0.97 ab	7.50 ± 1.15 a	6.52 ± 1.62 ab
3-Octen-2-one	9.14 ± 1.85 b	3.52 ± 0.80 c	3.46 ± 0.88 c	4.24 ± 0.18 c	16.69 ± 8.70 ab	19.00 ± 6.54 a
Benzeneacetaldehyde	41.30 ± 8.89 b	17.75 ± 4.12 c	20.47 ± 1.94 c	19.19 ± 2.02 c	69.59 ± 12.01 a	68.81 ± 14.17 a
β-Ocimene	29.34 ± 12.31 a	9.30 ± 2.02 b	8.11 ± 3.20 b	8.67 ± 1.65 b	7.67 ± 1.43 a	7.11 ± 1.07 a
1-Octanol	11.67 ± 1.96 b	9.64 ± 2.09 bc	5.10 ± 1.73 c	7.70 ± 1.06 c	21.96 ± 7.95 a	29.65 ± 13.48 a
Pyrazine, 3-ethyl-2,5-dimethyl-	5.56 ± 2.13 bc	3.25 ± 0.86 cd	2.89 ± 0.26 d	3.82 ± 1.51 cd	9.09 ± 4.19 ab	7.32 ± 2.28 ab
Nonanal	33.65 ± 8.91 c	38.11 ± 5.81 c	24.70 ± 4.14 d	30.16 ± 6.91 c	102.85 ± 21.17 b	181.85 ± 43.39 a
*trans*-2-Nonenal	3.03 ± 0.63 b	n.d.	1.67 ± 0.38 c	1.61 ± 0.37 c	5.06 ± 2.16 ab	8.38 ± 2.43 a
Decanal	17.33 ± 5.91 d	42.56 ± 20.61 bc	9.91 ± 2.34 e	26.24 ± 4.80 c	69.96 ± 20.91 b	169.55 ± 47.17 a
Undecanal	n.d.	1.33 ± 0.79 bc	0.73 ± 0.42 c	0.90 ± 0.37 c	2.97 ± 1.15 b	6.50 ± 2.58 a
Dodecanal	3.16 ± 0.44 a	1.86 ± 0.34 c	1.69 ± 0.52 cd	1.20 ± 0.20 d	2.41 ± 0.26 b	1.34 ± 0.49 cd

^1^ Data are shown as mean ± standard deviation. Letters in the row (a, b, c, d, and e) denote homogenous groups (Tukey test, α = 0.05). Abbreviations are as follows: 0D—hummus produced from unmalted chickpea seeds; 1D—hummus produced from chickpea malt germinated for 24 h; 2D—hummus produced from chickpea malt germinated for 48 h; 3D—hummus produced from chickpea malt germinated for 72 h; 4D—hummus produced from chickpea malt germinated for 96 h; 5D—hummus produced from chickpea malt germinated for 120 h. n.d. stands for ‘not detected’.

**Table 3:** Limits of detection (LOD) and limits of quantitation (LOQ) of compounds analyzed by GC/MS, utilizing internal standard method.

Compound	LOD [ppb]	LOQ [ppb]
1-Hexanol	43.72	132.5
2-Heptanone	1.57	4.77
Heptanal	1.95	5.91
Pyrazine, 2,5- dimethyl-	2.02	6.13
Benzaldehyde	7.4	22.44
1-Heptanol	1.9	5.77
1-Octen-3-ol	2.55	7.74
5-Hepten-2-one, 6-methyl-	5.15	15.6
Furan, 2-pentyl	4.97	15.08
Pyrazine, 2-ethyl-3-methyl-	3.46	10.49
Octanal	5.73	17.35
1-Hexanol, 2-ethyl	3.81	11.56
3-Octen-2-one	0.53	1.61
Benzeneacetaldehyde	17.07	51.71
β-Ocimene	4.94	14.97
1-Octanol	3.22	9.77
Pyrazine, 3-ethyl-2,5-dimethyl-	0.4	1.2
Nonanal	12.52	37.95
*trans*-2-Nonenal	0.24	0.73
Decanal	3.96	11.99
Undecanal	0.65	1.96
Dodecanal	0.27	0.81

## Data Availability

Data are available from the corresponding author upon reasonable request.

## Supplementary Materials

The following supporting information can be downloaded at: https://www.mdpi.com/article/10.3390/molecules30061231/s1, Mass spectra of the identified compounds, as well as sample chromatograms are provided in the Supplementary Materials. Figure S1. Mass spectrum of the compound with retention time 4.534 min (Hexanol); Figure S2. Mass spectrum of the compound with retention time 4.894 min (2-Heptanone); Figure S3. Mass spectrum of the compound with retention time 5.126 min (Heptanal); Figure S4. Mass spectrum of the compound with retention time 5.314 min (Pyrazine, 2,5-dimethyl-); Figure S5. Mass spectrum of the compound with retention time 6.254 min (Benzaldehyde); Figure S6. Mass spectrum of the compound with retention time 6.424 min (1-Heptanol); Figure S7. Mass spectrum of the compound with retention time 6.617 min (1-Octen-3-ol); Figure S8. Mass spectrum of the compound with retention time 6.700 min (5-Hepten-2-one, 6-methyl-); Figure S9. Mass spectrum of the compound with retention time 6.809 min (Furan, 2-pentyl-); Figure S10. Mass spectrum of the compound with retention time 7.020 min (Pyrazine, 2-ethyl-3-methyl-); Figure S11. Mass spectrum of the compound with retention time 7.064 min (Octanal); Figure S12. Mass spectrum of the compound with retention time 7.550 min (1-Hexanol, 2-ethyl-); Figure S13. Mass spectrum of the compound with retention time 7.723 min (3-Octen-2-one); Figure S14. Mass spectrum of the compound with retention time 7.843 min (Benzeneacetaldehyde); Figure S15. Mass spectrum of the compound with retention time 7.914 min (.beta.-Ocimene); Figure S16. Mass spectrum of the compound with retention time 8.374 min (1-Octanol); Figure S17. Mass spectrum of the compound with retention time 8.468 min (Pyrazine, 3-ethyl-2,5-dimethyl-); Figure S18. Mass spectrum of the compound with retention time 9.037 min (Nonanal); Figure S19. Mass spectrum of the compound with retention time 10.075 min (trans-2-Nonenal); Figure S20. Mass spectrum of the compound with retention time 10.944 min (Decanal); Figure S21. Mass spectrum of the compound with retention time 12.742 min (Undecanal); Figure S22. Mass spectrum of the compound with retention time 14.457 min (Dodecanal); Figure S23. Mass spectrum of the internal standard (2-Undecanone); Figure S24. Representative chromatogram of the 0D sample; Figure S25. Representative chromatogram of the 1D sample; Figure S26. Representative chromatogram of the 2D sample; Figure S27. Representative chromatogram of the 3D sample; Figure S28. Representative chromatogram of the 4D sample; Figure S29. Representative chromatogram of the 5D sample.
