# Supplementary material for: Influence of Malted Chickpea on the Composition of Volatiles in Hummus
Source: Molecules. 2025 Mar 10;30(6):1231. doi: 10.3390/molecules30061231 (PMC11944303; doi:10.3390/molecules30061231)
Supplement: Supplementary file 1 [file molecules-30-01231-s001.zip › molecules-3492423-supplementary.pdf]

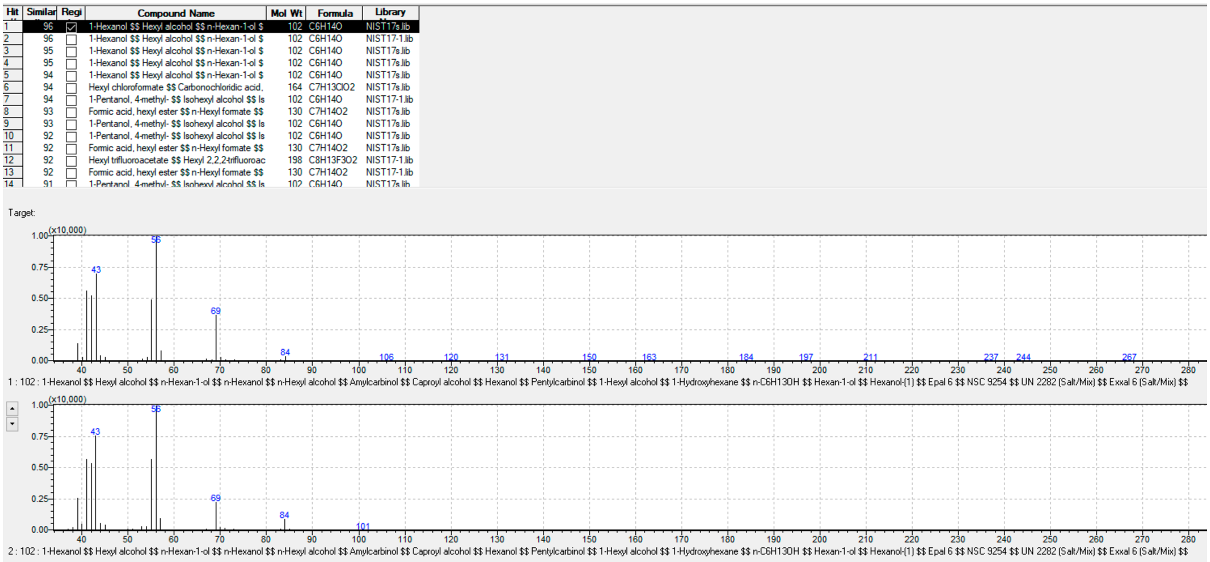

Hit	Similar	Regi	Compound Name	Mol Wt	Formula	Library
1	96	☑	1-Hexanol $$ Hexyl alcohol $$ n-Hexan-1-ol $	102	C6H14O	NIST17s.lib
2	96	☐	1-Hexanol $$ Hexyl alcohol $$ n-Hexan-1-ol $	102	C6H14O	NIST17-1.lib
3	95	☐	1-Hexanol $$ Hexyl alcohol $$ n-Hexan-1-ol $	102	C6H14O	NIST17s.lib
4	95	☐	1-Hexanol $$ Hexyl alcohol $$ n-Hexan-1-ol $	102	C6H14O	NIST17s.lib
5	94	☐	1-Hexanol $$ Hexyl alcohol $$ n-Hexan-1-ol $	102	C6H14O	NIST17s.lib
6	94	☐	Hexyl chloroformate $$ Carbonochloridic acid,	164	C7H13ClO2	NIST17s.lib
7	94	☐	1-Pentanol, 4-methyl- $$ Isohexyl alcohol $$ Is	102	C6H14O	NIST17-1.lib
8	93	☐	Formic acid, hexyl ester $$ n-Hexyl formate $$	130	C7H14O2	NIST17s.lib
9	93	☐	1-Pentanol, 4-methyl- $$ Isohexyl alcohol $$ Is	102	C6H14O	NIST17s.lib
10	92	☐	1-Pentanol, 4-methyl- $$ Isohexyl alcohol $$ Is	102	C6H14O	NIST17s.lib
11	92	☐	Formic acid, hexyl ester $$ n-Hexyl formate $$	130	C7H14O2	NIST17s.lib
12	92	☐	Hexyl trifluoroacetate $$ Hexyl 2,2,2-trifluoroac	198	C8H13F3O2	NIST17-1.lib
13	92	☐	Formic acid, hexyl ester $$ n-Hexyl formate $$	130	C7H14O2	NIST17-1.lib
14	91	☐	1-Pentanol, 4-methyl- $$ Isohexyl alcohol $$ Is	102	C6H14O	NIST17s.lib

Figure S1. Mass spectrum of the compound with retention time 4.534 min (Hexanol)

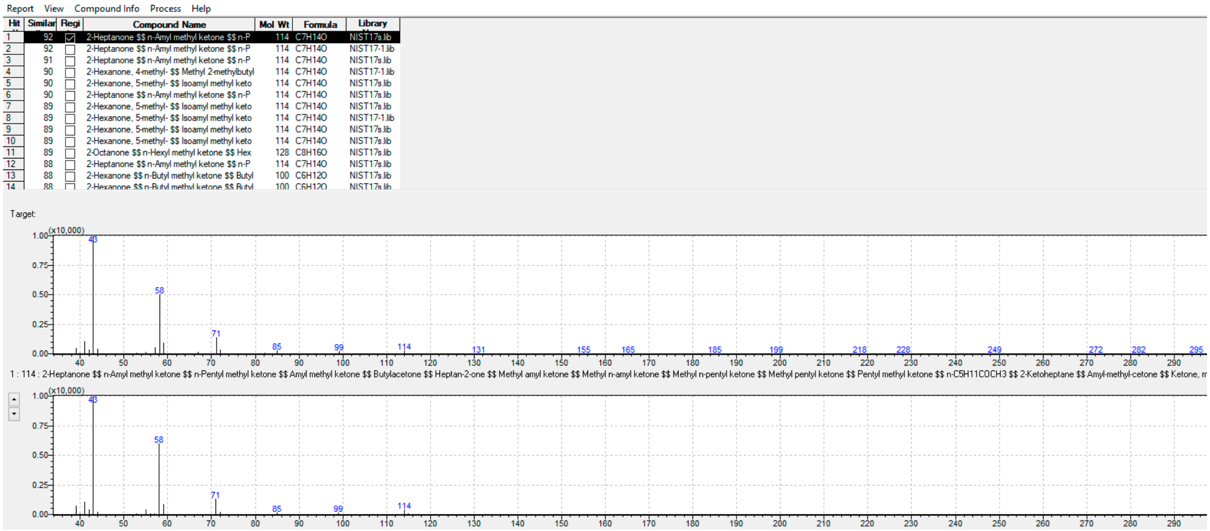

Hit	Similar	Regi	Compound Name	Mol Wt	Formula	Library
1	92	☑	2-Heptanone $$ n-Amyl methyl ketone $$ n-P	114	C7H14O	NIST17s.lib
2	92	☐	2-Heptanone $$ n-Amyl methyl ketone $$ n-P	114	C7H14O	NIST17-1.lib
3	91	☐	2-Heptanone $$ n-Amyl methyl ketone $$ n-P	114	C7H14O	NIST17s.lib
4	90	☐	2-Hexanone, 4-methyl- $$ Methyl 2-methylbutyl	114	C7H14O	NIST17-1.lib
5	90	☐	2-Hexanone, 5-methyl- $$ Isoamyl methyl keto	114	C7H14O	NIST17s.lib
6	90	☐	2-Heptanone $$ n-Amyl methyl ketone $$ n-P	114	C7H14O	NIST17s.lib
7	89	☐	2-Hexanone, 5-methyl- $$ Isoamyl methyl keto	114	C7H14O	NIST17s.lib
8	89	☐	2-Hexanone, 5-methyl- $$ Isoamyl methyl keto	114	C7H14O	NIST17-1.lib
9	89	☐	2-Hexanone, 5-methyl- $$ Isoamyl methyl keto	114	C7H14O	NIST17s.lib
10	89	☐	2-Hexanone, 5-methyl- $$ Isoamyl methyl keto	114	C7H14O	NIST17s.lib
11	89	☐	2-Octanone $$ n-Hexyl methyl ketone $$ Hex	128	C8H16O	NIST17s.lib
12	88	☐	2-Heptanone $$ n-Amyl methyl ketone $$ n-P	114	C7H14O	NIST17s.lib
13	88	☐	2-Hexanone $$ n-Butyl methyl ketone $$ Butyl	100	C6H12O	NIST17s.lib
14	88	☐	2-Hexanone $$ n-Butyl methyl ketone $$ Butyl	100	C6H12O	NIST17s.lib

Figure S2. Mass spectrum of the compound with retention time 4.894 min (2-Heptanone)

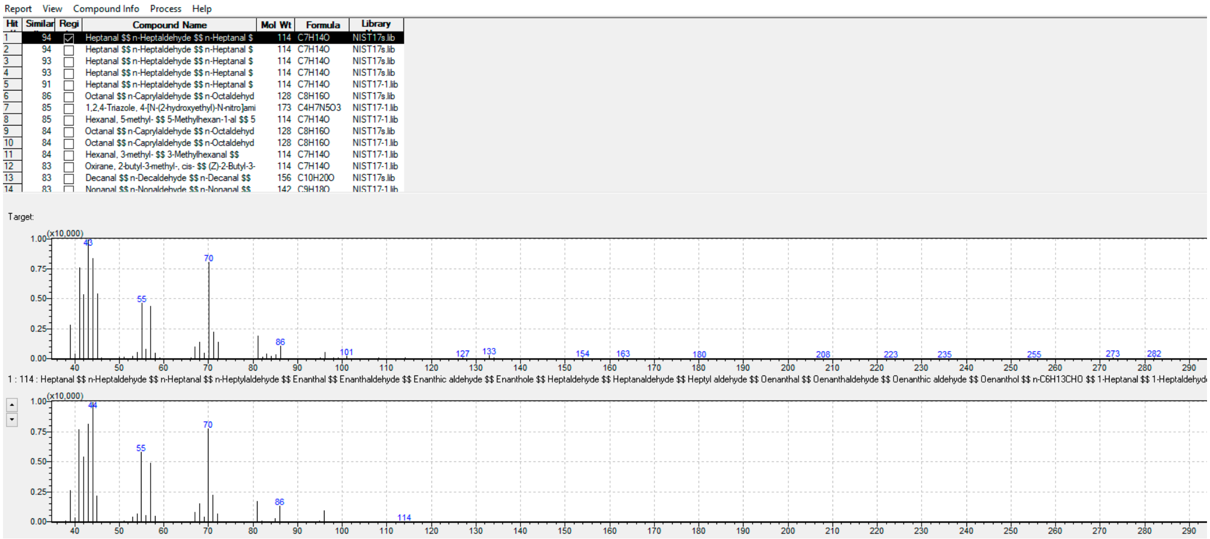

Figure S3. Mass spectrum of the compound with retention time 5.126 min (Heptanal)

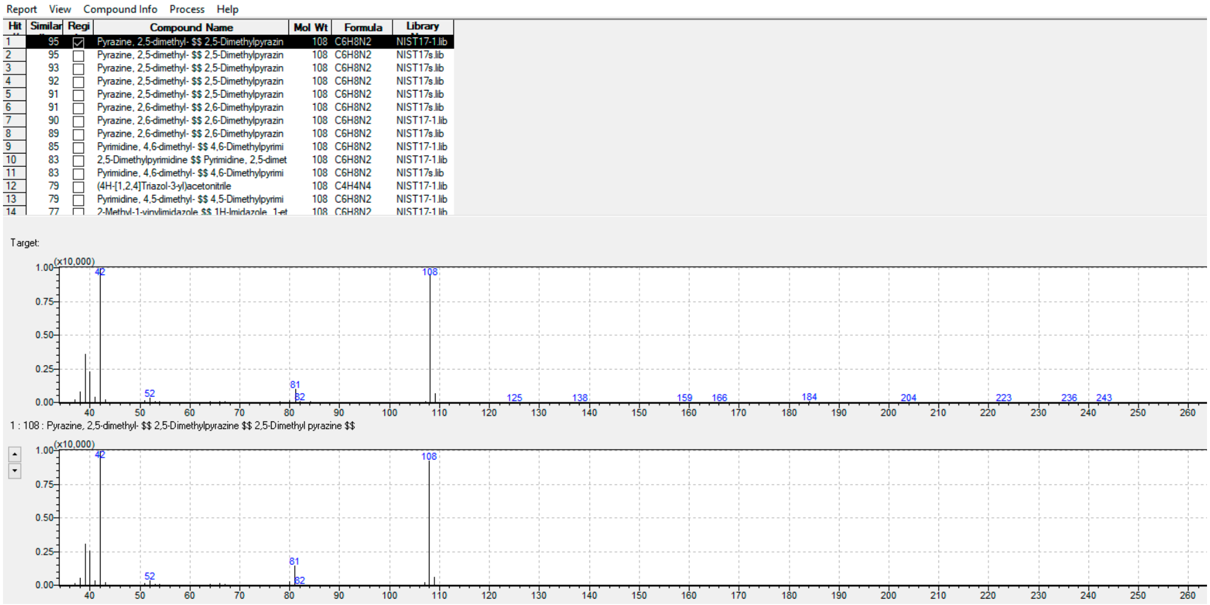

Figure S4. Mass spectrum of the compound with retention time 5.314 min (Pyrazine, 2,5-dimethyl-)

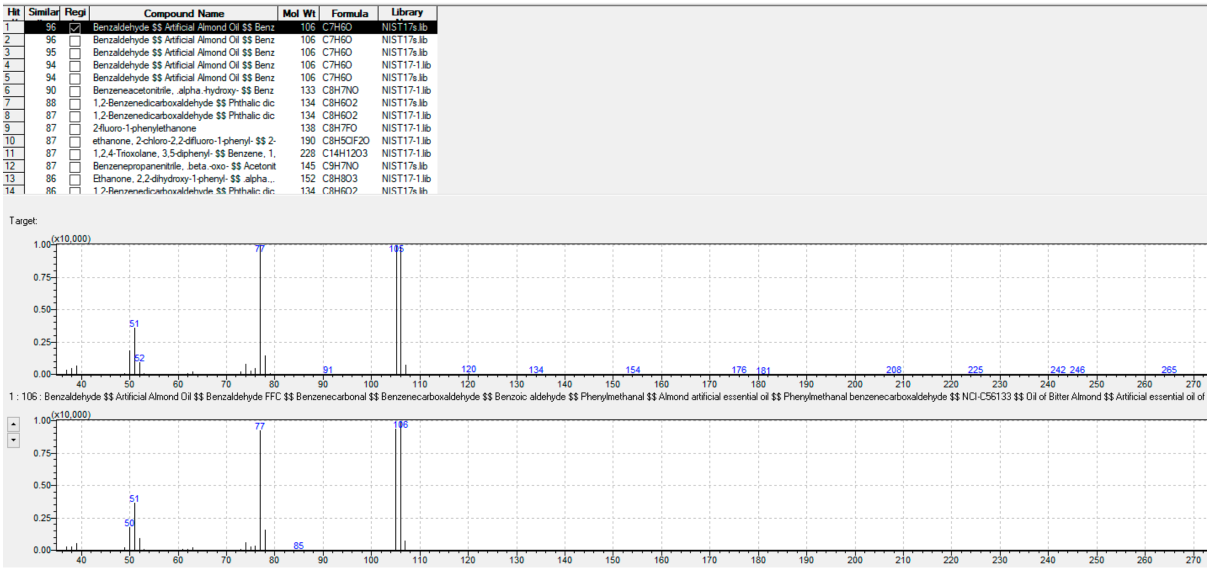

Figure S5. Mass spectrum of the compound with retention time 6.254 min (Benzaldehyde)

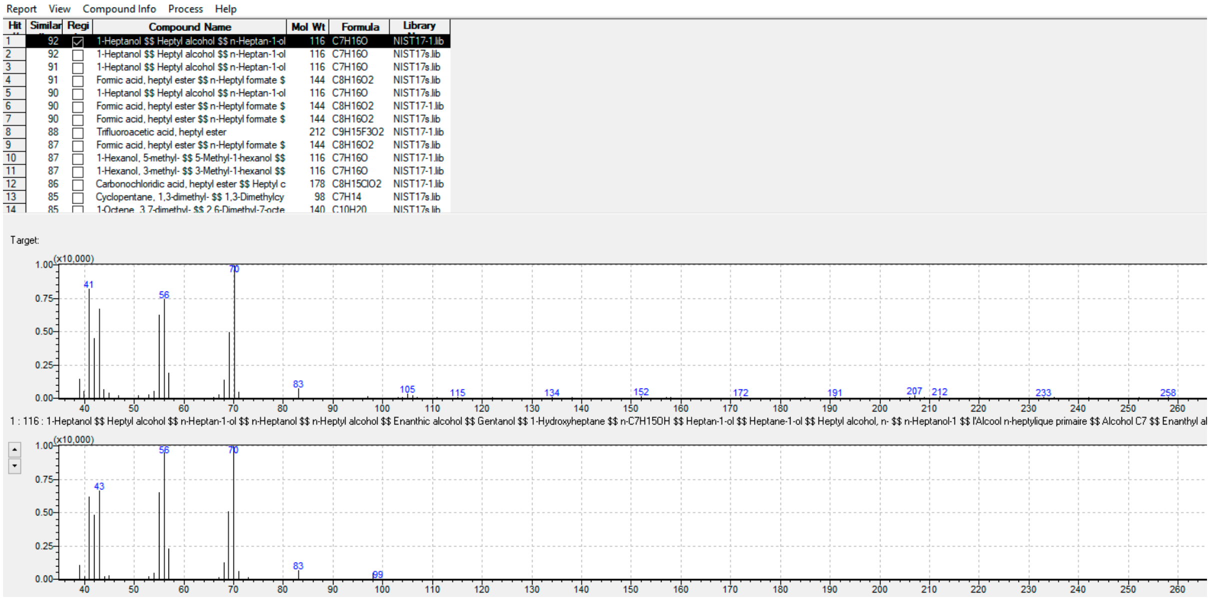

Figure S6. Mass spectrum of the compound with retention time 6.424 min (1-Heptanol)

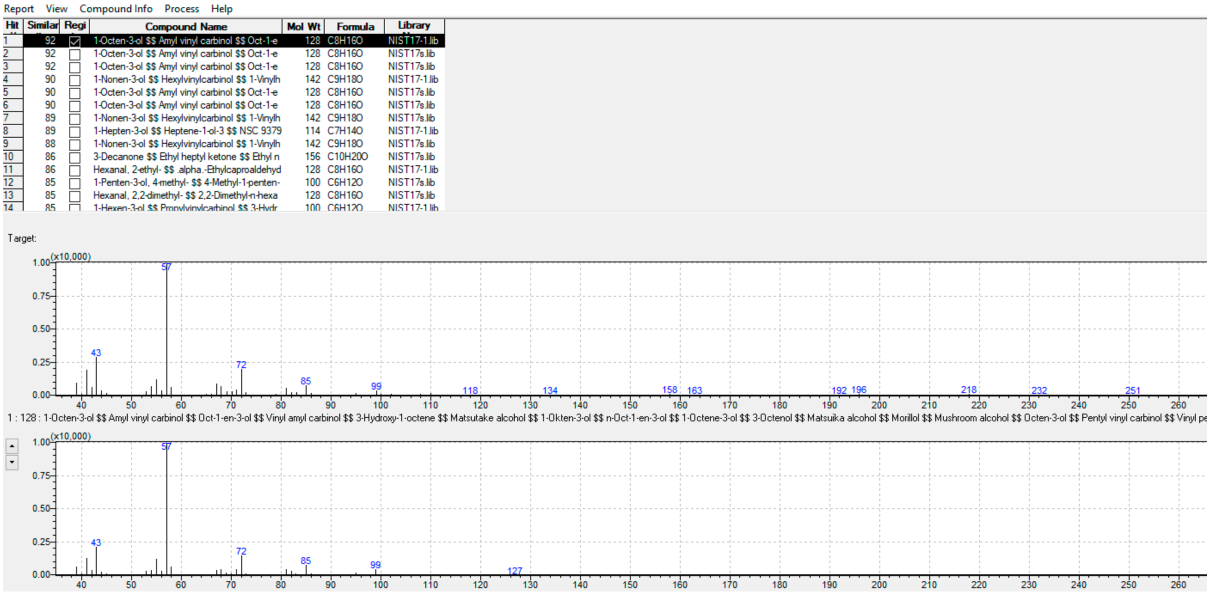

Figure S7. Mass spectrum of the compound with retention time 6.617 min (1-Octen-3-ol)

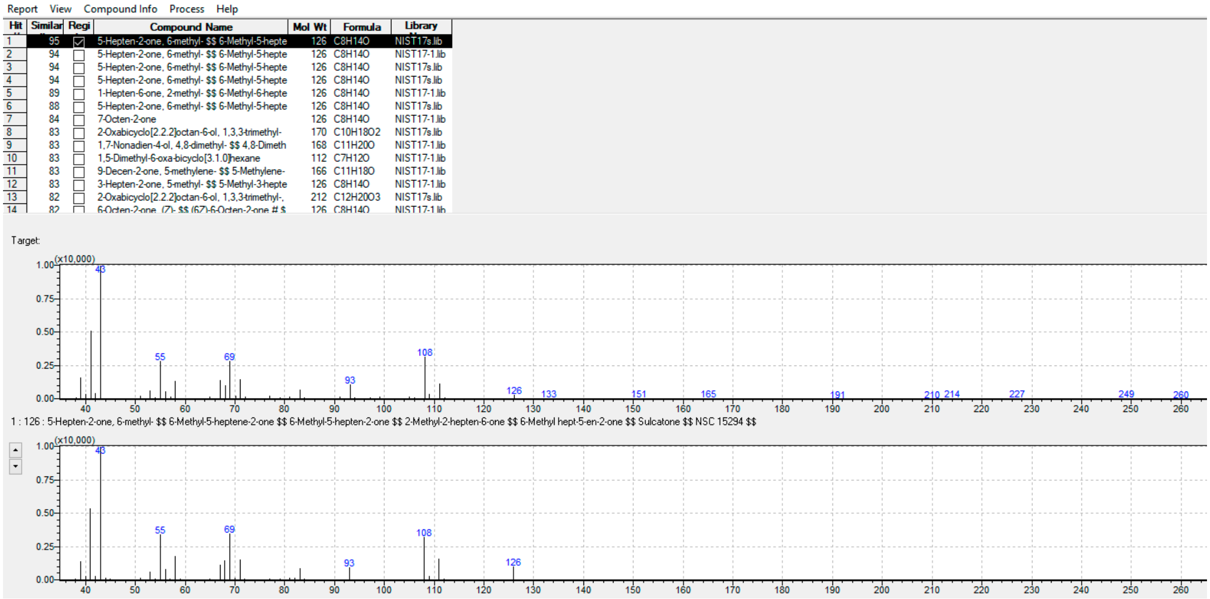

Figure S8. Mass spectrum of the compound with retention time 6.700 min (5-Hepten-2-one, 6-methyl-)

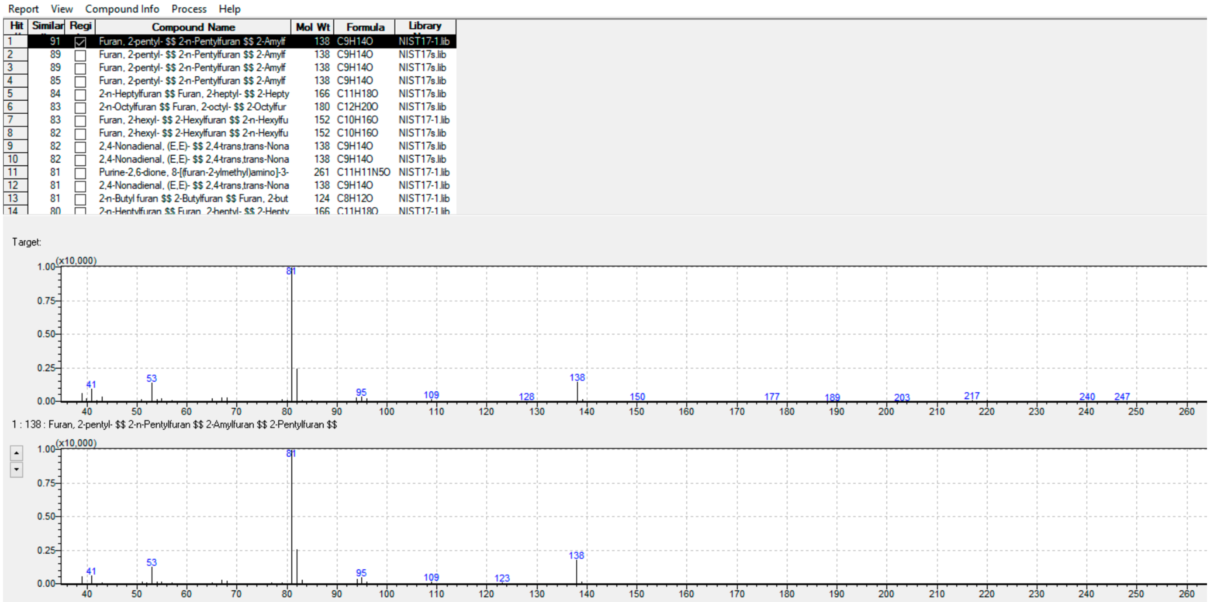

Figure S9. Mass spectrum of the compound with retention time 6.809 min (Furan, 2-pentyl-)

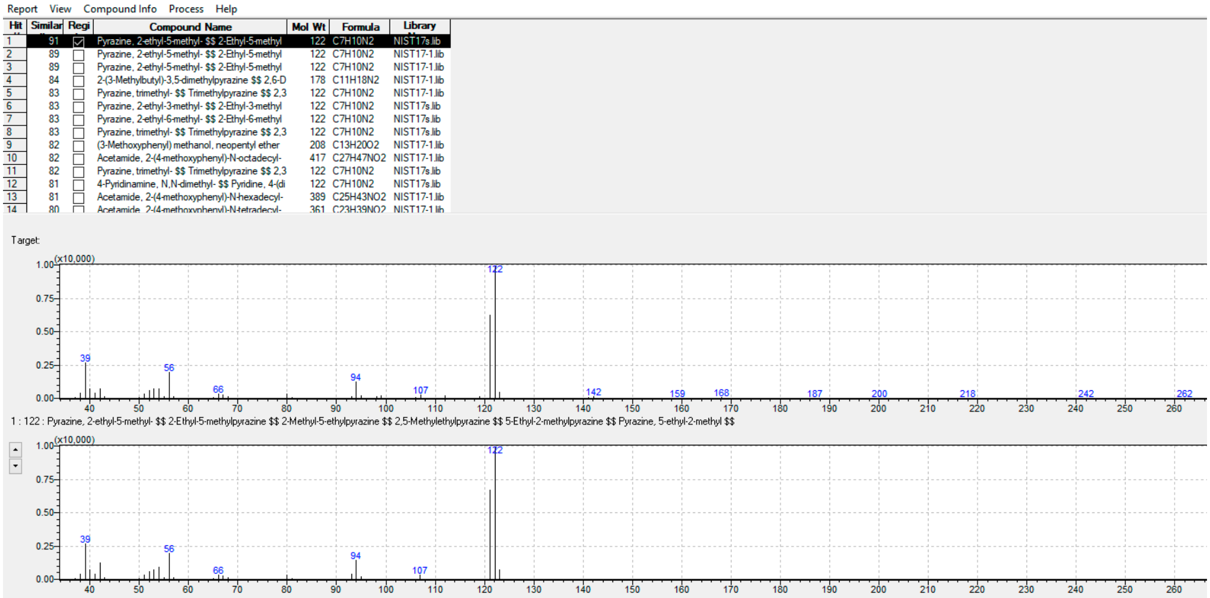

Figure S10. Mass spectrum of the compound with retention time 7.020 min (Pyrazine, 2-ethyl-3-methyl-)

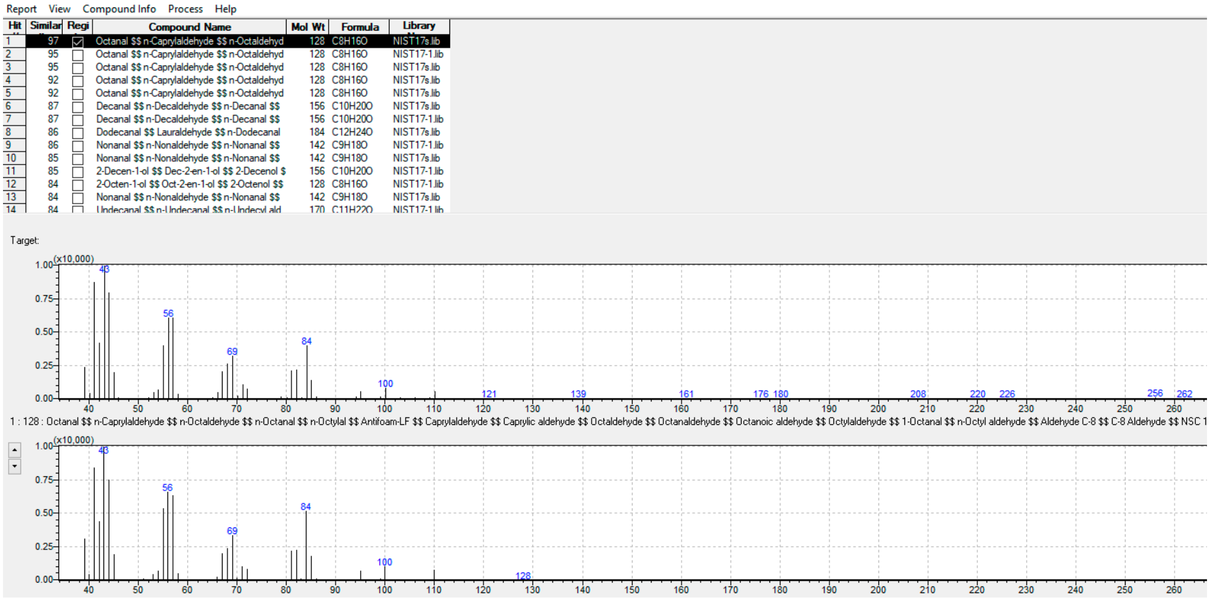

Figure S11. Mass spectrum of the compound with retention time 7.064 min (Octanal)

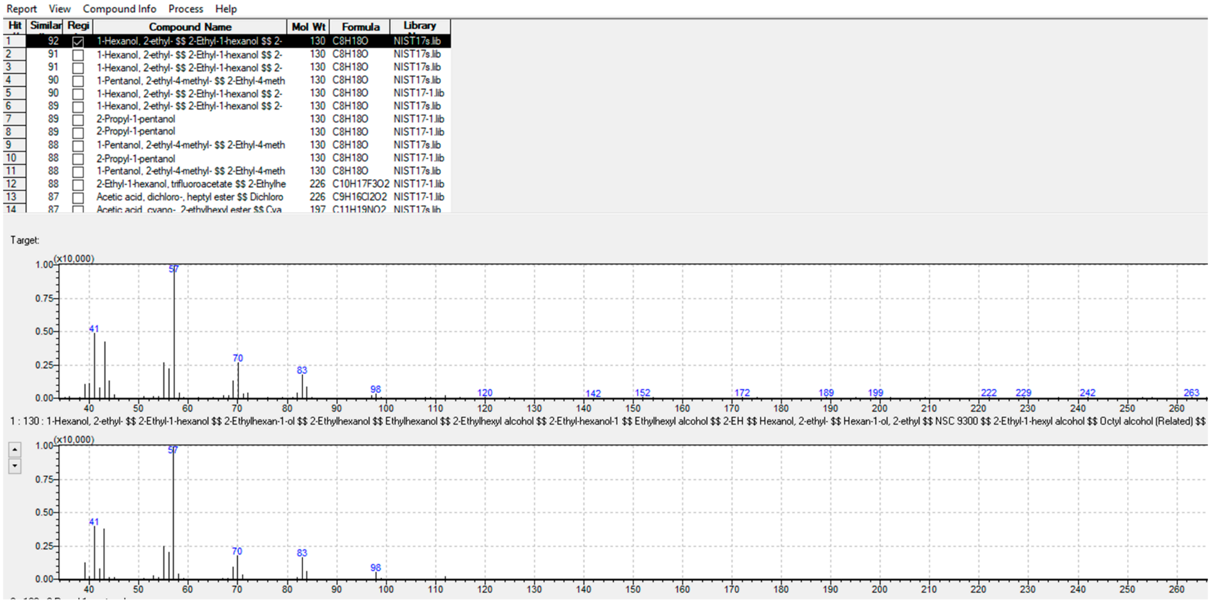

Figure S12. Mass spectrum of the compound with retention time 7.550 min (1-Hexanol, 2-ethyl-)

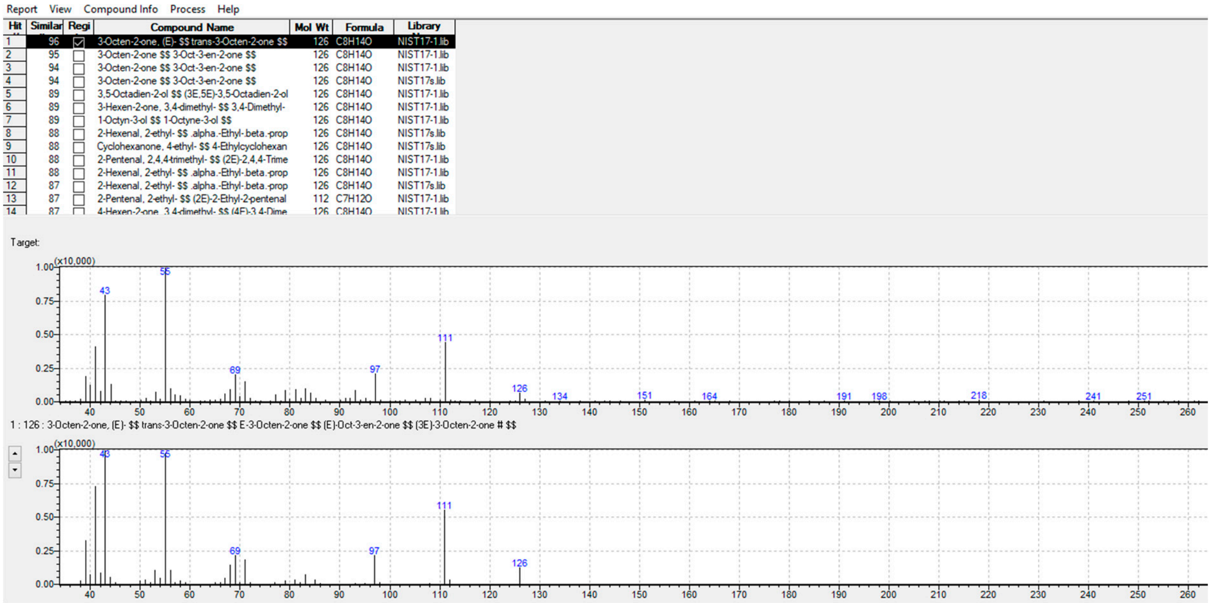
Report View Compound Info Process Help

Hit	Similar	Regi	Compound Name	Mol Wt	Formula	Library
1	96	☑	3-Octen-2-one, (E)- $$ trans-3-Octen-2-one $$	126	C8H14O	NIST17-1.lib
2	95	☐	3-Octen-2-one $$ 3-Oct-3-en-2-one $$	126	C8H14O	NIST17-1.lib
3	94	☐	3-Octen-2-one $$ 3-Oct-3-en-2-one $$	126	C8H14O	NIST17-1.lib
4	94	☐	3-Octen-2-one $$ 3-Oct-3-en-2-one $$	126	C8H14O	NIST17s.lib
5	89	☐	3,5-Octadien-2-ol $$ (3E,5E)-3,5-Octadien-2-ol	126	C8H14O	NIST17-1.lib
6	89	☐	3-Hexen-2-one, 3,4-dimethyl- $$ 3,4-Dimethyl-	126	C8H14O	NIST17-1.lib
7	89	☐	1-Octyn-3-ol $$ 1-Octyne-3-ol $$	126	C8H14O	NIST17-1.lib
8	88	☐	2-Hexenal, 2-ethyl- $$ .alpha.-Ethyl-.beta.-prop	126	C8H14O	NIST17s.lib
9	88	☐	Cyclohexanone, 4-ethyl- $$ 4-Ethylcyclohexan	126	C8H14O	NIST17s.lib
10	88	☐	2-Pentenal, 2,4,4-trimethyl- $$ (2E)-2,4,4-Trime	126	C8H14O	NIST17-1.lib
11	88	☐	2-Hexenal, 2-ethyl- $$ .alpha.-Ethyl-.beta.-prop	126	C8H14O	NIST17-1.lib
12	87	☐	2-Hexenal, 2-ethyl- $$ .alpha.-Ethyl-.beta.-prop	126	C8H14O	NIST17s.lib
13	87	☐	2-Pentenal, 2-ethyl- $$ (2E)-2-Ethyl-2-pentenal	112	C7H12O	NIST17-1.lib
14	87	☐	4-Hexen-2-one, 3,4-dimethyl- $$ (4E)-3,4-Dime	126	C8H14O	NIST17-1.lib

Figure S13. Mass spectrum of the compound with retention time 7.723 min (3-Octen-2-one)

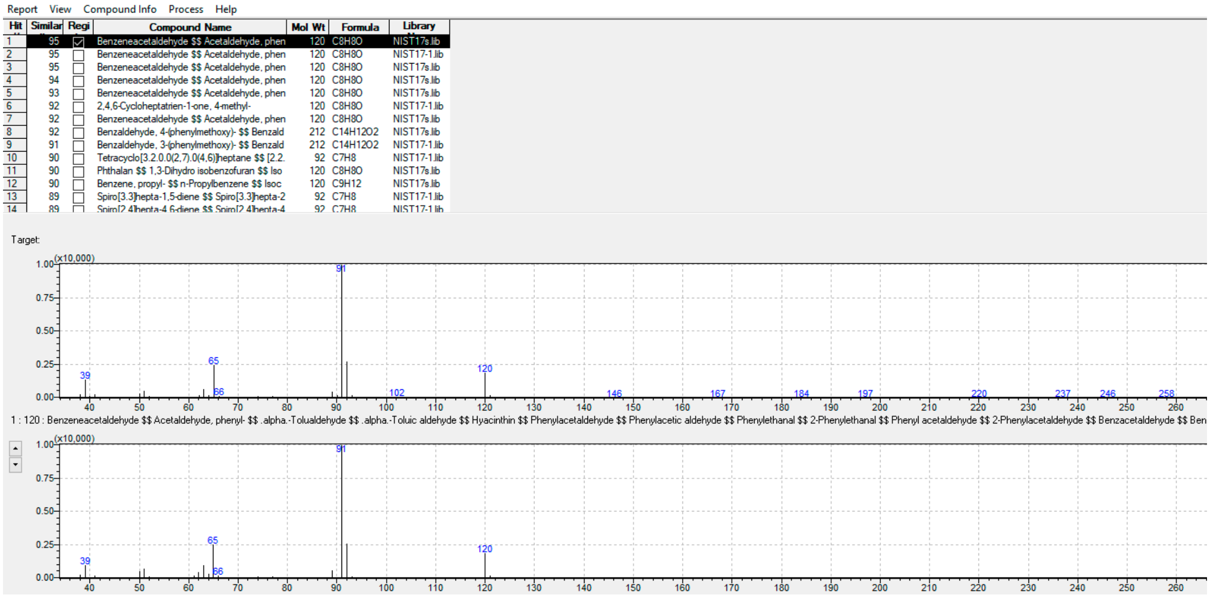
Report View Compound Info Process Help

Hit	Similar	Regi	Compound Name	Mol Wt	Formula	Library
1	95	☑	Benzeneacetaldehyde $$ Acetaldehyde, phen	120	C8H8O	NIST17s.lib
2	95	☐	Benzeneacetaldehyde $$ Acetaldehyde, phen	120	C8H8O	NIST17-1.lib
3	95	☐	Benzeneacetaldehyde $$ Acetaldehyde, phen	120	C8H8O	NIST17s.lib
4	94	☐	Benzeneacetaldehyde $$ Acetaldehyde, phen	120	C8H8O	NIST17s.lib
5	93	☐	Benzeneacetaldehyde $$ Acetaldehyde, phen	120	C8H8O	NIST17s.lib
6	92	☐	2,4,6-Cycloheptatrien-1-one, 4-methyl-	120	C8H8O	NIST17-1.lib
7	92	☐	Benzeneacetaldehyde $$ Acetaldehyde, phen	120	C8H8O	NIST17s.lib
8	92	☐	Benzaldehyde, 4-(phenylmethoxy)- $$ Benzald	212	C14H12O2	NIST17s.lib
9	91	☐	Benzaldehyde, 3-(phenylmethoxy)- $$ Benzald	212	C14H12O2	NIST17-1.lib
10	90	☐	Tetracyclo[3.2.0.0(2,7).0(4,6)]heptane $$ [2.2.	92	C7H8	NIST17-1.lib
11	90	☐	Phthalan $$ 1,3-Dihydro isobenzofuran $$ Iso	120	C8H8O	NIST17s.lib
12	90	☐	Benzene, propyl- $$ n-Propylbenzene $$ Isoc	120	C9H12	NIST17s.lib
13	89	☐	Spiro[3.3]hepta-1,5-diene $$ Spiro[3.3]hepta-2	92	C7H8	NIST17-1.lib
14	89	☐	Spiro[2.4]hepta-4,6-diene $$ Spiro[2.4]hepta-4	92	C7H8	NIST17-1.lib

Figure S14. Mass spectrum of the compound with retention time 7.843 min (Benzeneacetaldehyde)

Report View Compound Info Process Help

Hit	Similar	Regi	Compound Name	Mol Wt	Formula	Library
1	95	☑	.beta.-Ocimene $$ 1,3,6-Octatriene, 3,7-dimet	136	C10H16	NIST17-1.lib
2	95	☐	1,3,6-Octatriene, 3,7-dimethyl-, (Z)- $$ .beta.-ci	136	C10H16	NIST17s.lib
3	93	☐	1,3,7-Octatriene, 3,7-dimethyl- $$ 2,6-Dimethyl	136	C10H16	NIST17-1.lib
4	93	☐	1,3,6-Octatriene, 3,7-dimethyl-, (Z)- $$ .beta.-ci	136	C10H16	NIST17s.lib
5	92	☐	trans-.beta.-Ocimene $$ 1,3,6-Octatriene, 3,7-	136	C10H16	NIST17s.lib
6	92	☐	trans-.beta.-Ocimene $$ 1,3,6-Octatriene, 3,7-	136	C10H16	NIST17s.lib
7	92	☐	1,3,6-Octatriene, 3,7-dimethyl-, (Z)- $$ .beta.-ci	136	C10H16	NIST17-1.lib
8	91	☐	trans-.beta.-Ocimene $$ 1,3,6-Octatriene, 3,7-	136	C10H16	NIST17-1.lib
9	91	☐	Cyclopentene, 3-isopropenyl-5,5-dimethyl- $$ 5	136	C10H16	NIST17-1.lib
10	90	☐	Bicyclo[2.2.1]heptane, 2,2-dimethyl-3-methylen	136	C10H16	NIST17-1.lib
11	90	☐	3-Carene $$ Bicyclo[4.1.0]hept-3-ene, 3,7,7-tri	136	C10H16	NIST17s.lib
12	90	☐	1,3,7-Octatriene, 3,7-dimethyl- $$ 2,6-Dimethyl	136	C10H16	NIST17s.lib
13	90	☐	.alpha.-Pinene $$ Bicyclo[3.1.1]hept-2-ene, 2,	136	C10H16	NIST17-1.lib
14	89	☐	Bicyclo[2.2.1]heptane, 7,7-dimethyl-2-methylen	136	C10H16	NIST17s.lib

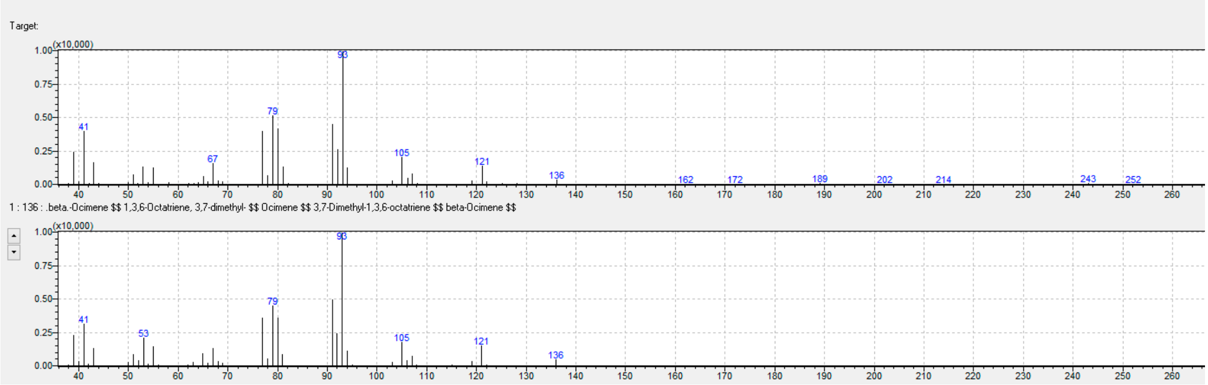

Figure S15. Mass spectrum of the compound with retention time 7.914 min (.beta.-Ocimene)

Report View Compound Info Process Help

Hit	Similar	Regi	Compound Name	Mol Wt	Formula	Library
1	92	☑	1-Octanol $$ Octyl alcohol $$ n-Octan-1-ol $$	130	C8H18O	NIST17s.lib
2	92	☐	1-Octanol $$ Octyl alcohol $$ n-Octan-1-ol $$	130	C8H18O	NIST17s.lib
3	91	☐	1-Nonanol $$ Nonyl alcohol $$ n-Nonyl alcoh	144	C9H20O	NIST17s.lib
4	91	☐	1-Octanol $$ Octyl alcohol $$ n-Octan-1-ol $$	130	C8H18O	NIST17-1.lib
5	90	☐	1-Nonanol $$ Nonyl alcohol $$ n-Nonyl alcoh	144	C9H20O	NIST17s.lib
6	90	☐	1-Octanol $$ Octyl alcohol $$ n-Octan-1-ol $$	130	C8H18O	NIST17s.lib
7	90	☐	Formic acid, octyl ester $$ n-Octyl formate $$	158	C9H18O2	NIST17s.lib
8	90	☐	1-Octyl trifluoroacetate $$ Octyl trifluoroacetat	226	C10H17F3O2	NIST17s.lib
9	90	☐	Cyclopropane, octyl- $$ Octane, 1-cyclopropyl	154	C11H22	NIST17-1.lib
10	90	☐	Cyclopropane, 1-heptyl-2-methyl- $$ 1-Heptyl-2	154	C11H22	NIST17-1.lib
11	90	☐	Chloromethyl octyl ether $$ 1-(Chloromethoxy)	178	C9H19ClO	NIST17-1.lib
12	89	☐	1-Decanol $$ Decyl alcohol $$ n-Decan-1-ol $	158	C10H22O	NIST17s.lib
13	89	☐	1-Octanol $$ Octyl alcohol $$ n-Octan-1-ol $$	130	C8H18O	NIST17s.lib
14	89	☐	1-Nonanol $$ Nonyl alcohol $$ n-Nonyl alcoh	144	C9H20O	NIST17s.lib

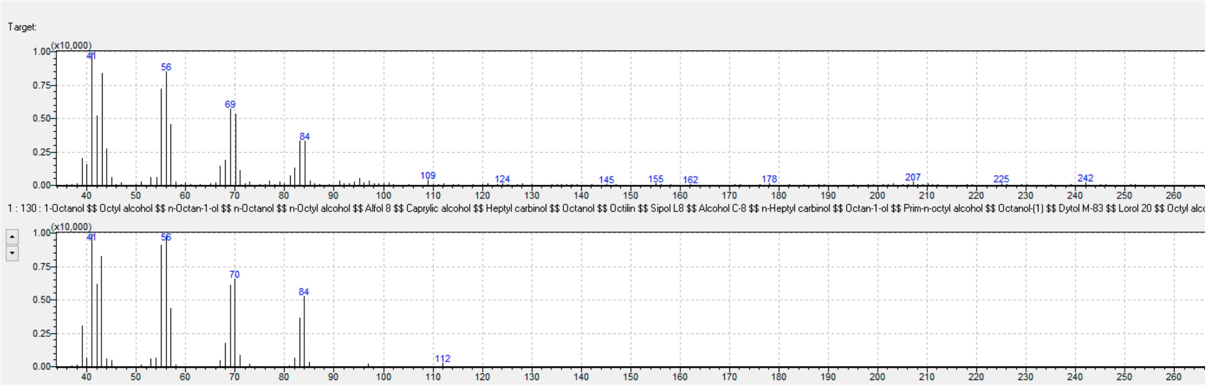

Figure S16. Mass spectrum of the compound with retention time 8.374 min (1-Octanol)

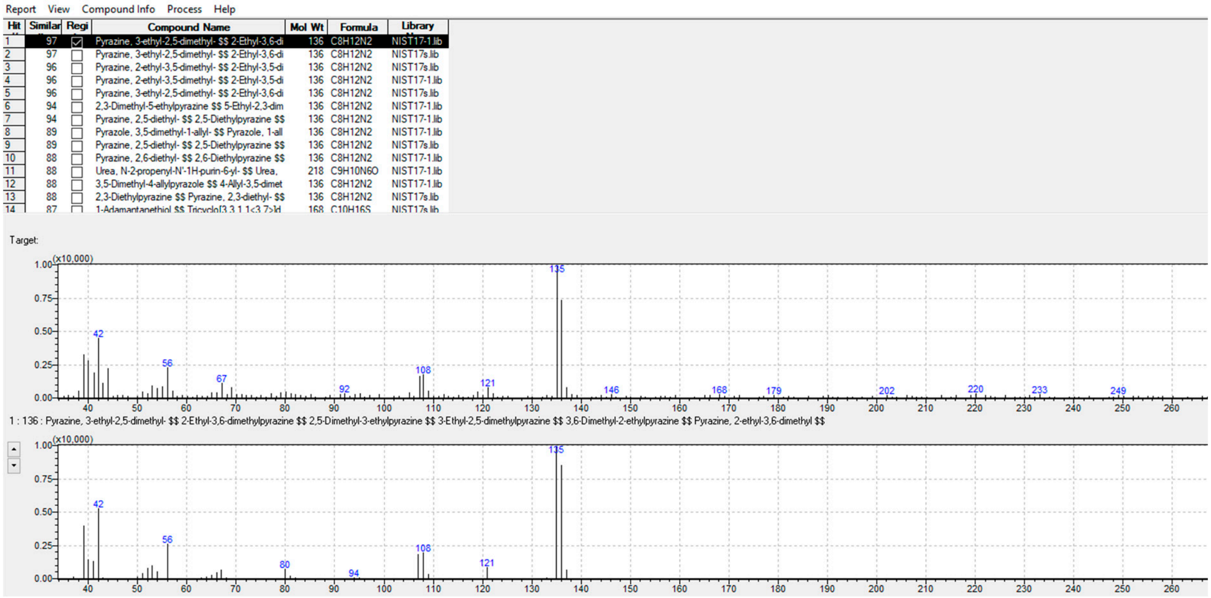

Figure S17. Mass spectrum of the compound with retention time 8.468 min (Pyrazine, 3-ethyl-2,5-dimethyl-)

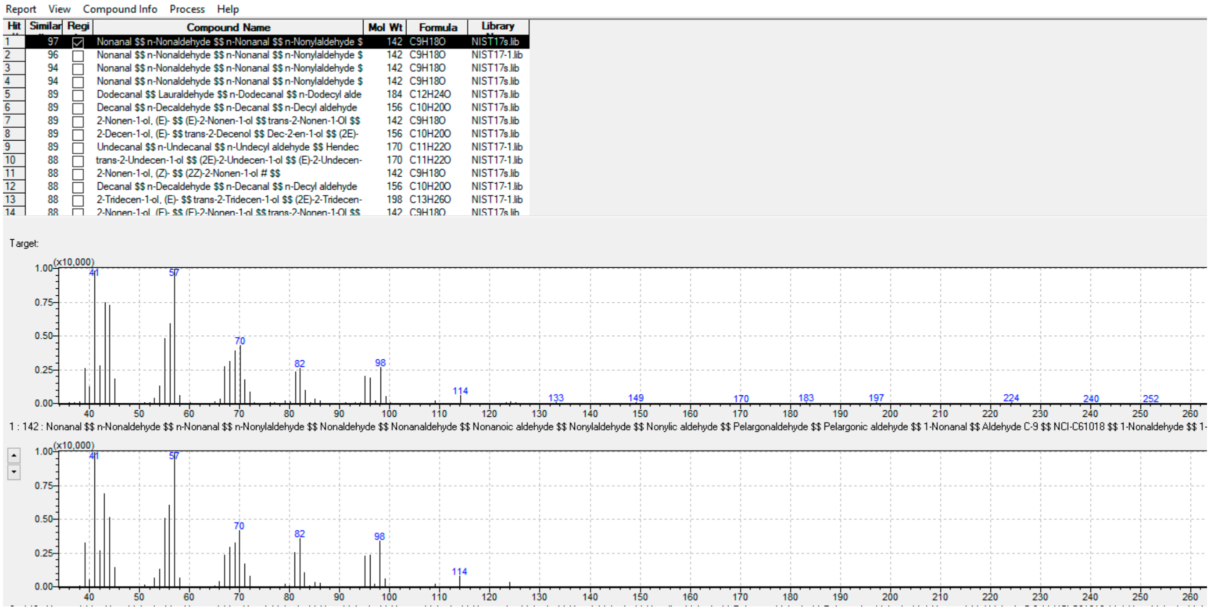

Figure S18. Mass spectrum of the compound with retention time 9.037 min (Nonanal)

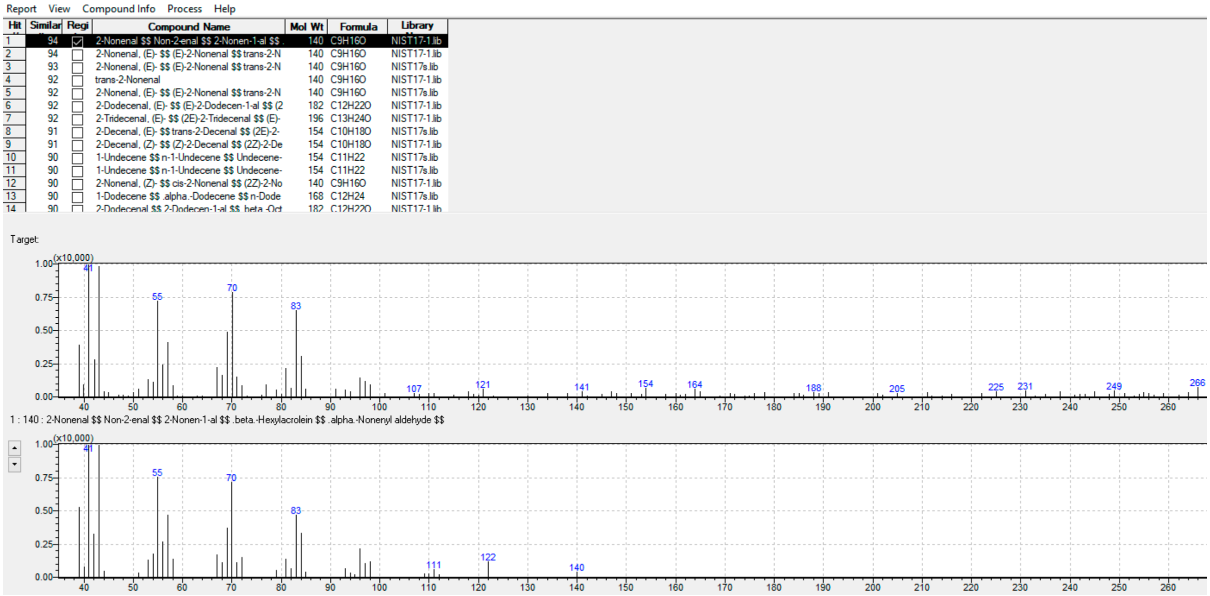

Figure S19. Mass spectrum of the compound with retention time 10.075 min (trans-2-Nonenal)

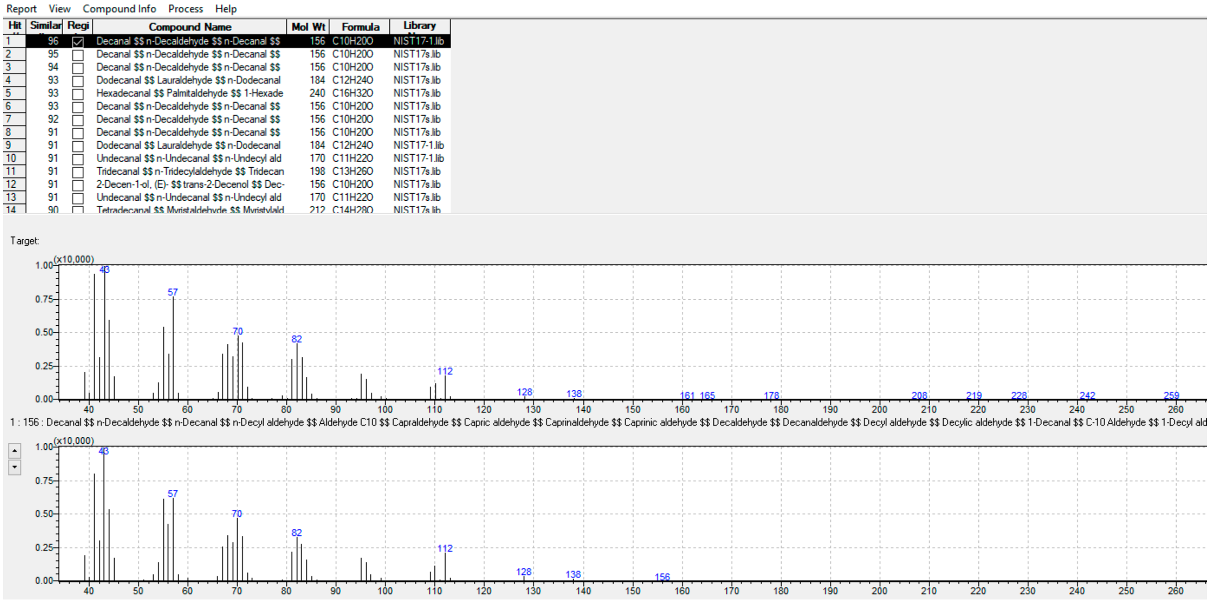

Figure S20. Mass spectrum of the compound with retention time 10.944 min (Decanal)

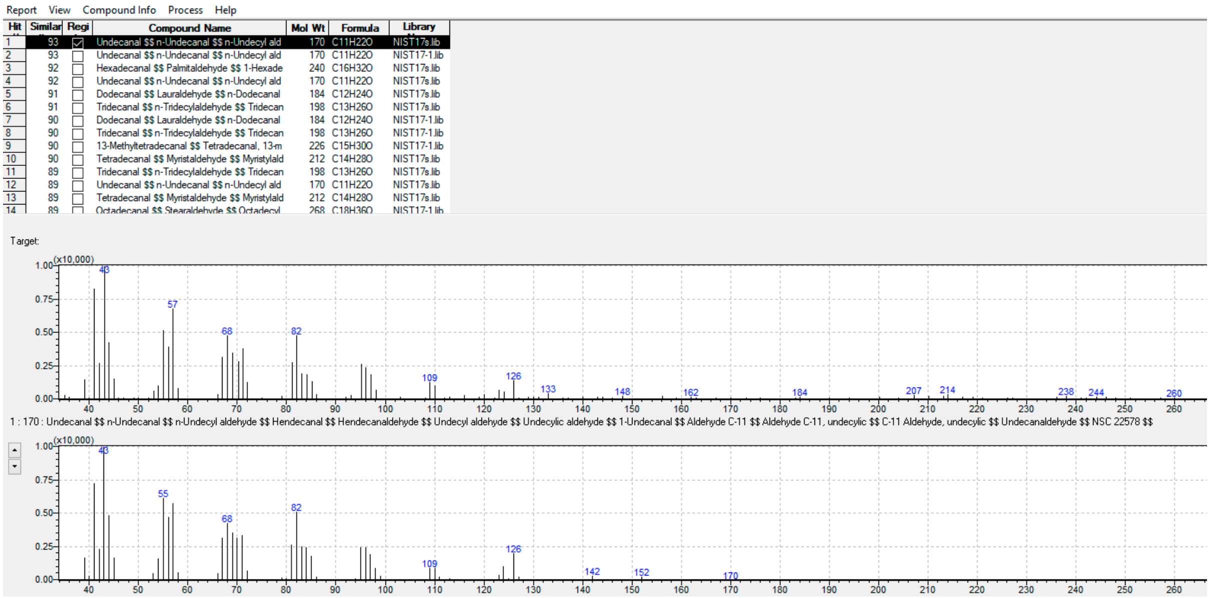

Figure S21. Mass spectrum of the compound with retention time 12.742 min (Undecanal)

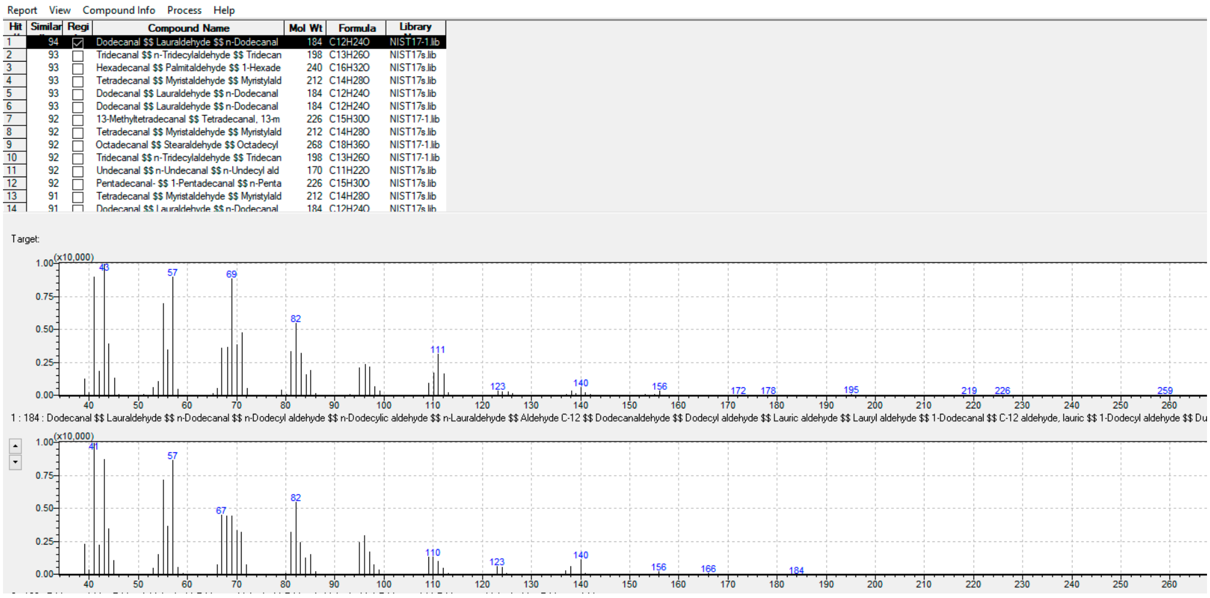

Figure S22. Mass spectrum of the compound with retention time 14.457 min (Dodecanal)

Report View Compound Info Process Help

Hit	Similar	Regi	Compound Name	Mol Wt	Formula	Library
1	97	☑	2-Undecanone $$ Ketone, methyl nonyl $$ M	170	C11H22O	NIST17s.lib
2	96	☐	2-Undecanone $$ Ketone, methyl nonyl $$ M	170	C11H22O	NIST17s.lib
3	94	☐	2-Undecanone $$ Ketone, methyl nonyl $$ M	170	C11H22O	NIST17s.lib
4	93	☐	2-Undecanone $$ Ketone, methyl nonyl $$ M	170	C11H22O	NIST17-1.lib
5	93	☐	2-Dodecanone $$ Decyl methyl ketone $$ Do	184	C12H24O	NIST17s.lib
6	93	☐	2-Tridecanone $$ Mathyl undecyl kepoje $$	198	C13H26O	NIST17s.lib
7	93	☐	2-Undecanone $$ Ketone, methyl nonyl $$ M	170	C11H22O	NIST17s.lib
8	93	☐	2-Decanone $$ Decan-2-one $$ Methyl octyl	156	C10H20O	NIST17-1.lib
9	92	☐	2-Dodecanone $$ Decyl methyl ketone $$ Do	184	C12H24O	NIST17-1.lib
10	92	☐	2-Pentadecanone $$ Methyl tridecyl ketone $	226	C15H30O	NIST17s.lib
11	91	☐	2-Dodecanone $$ Decyl methyl ketone $$ Do	184	C12H24O	NIST17s.lib
12	91	☐	2-Dodecanone $$ Decyl methyl ketone $$ Do	184	C12H24O	NIST17s.lib
13	91	☐	2-Tridecanone $$ Mathyl undecyl kepoje $$	198	C13H26O	NIST17s.lib
14	91	☐	2-Decanone $$ Decan-2-one $$ Methyl octyl	156	C10H20O	NIST17s.lib

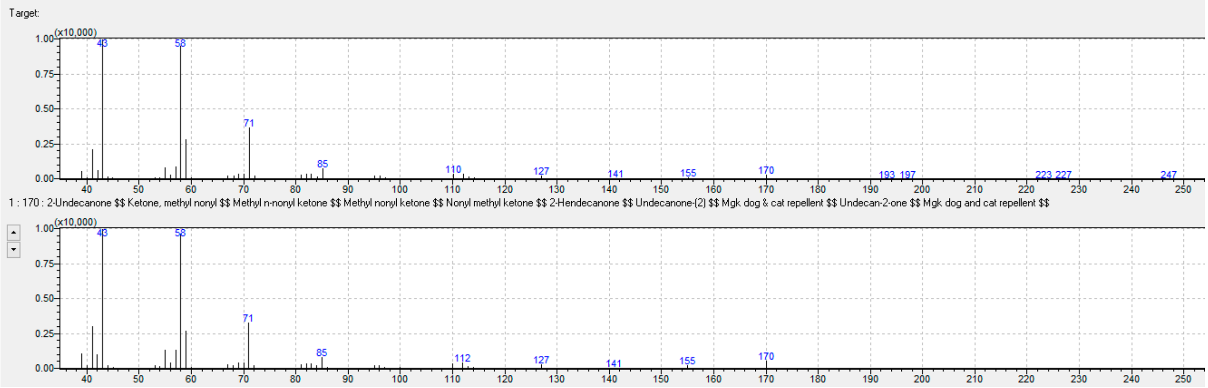

Figure S23. Mass spectrum of the internal standard (2-Undecanone)

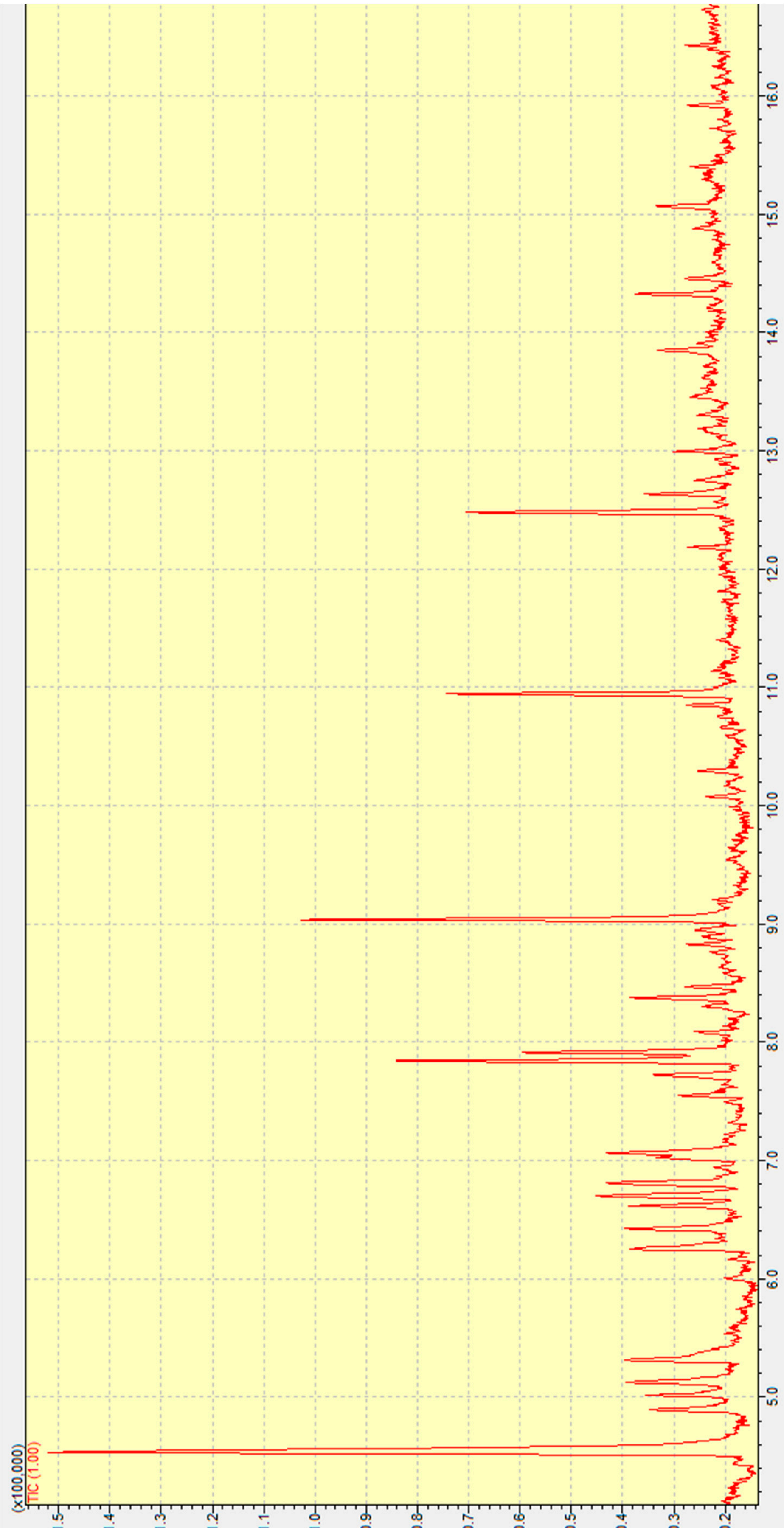

Figure S24. Representative chromatogram of the 0D sample

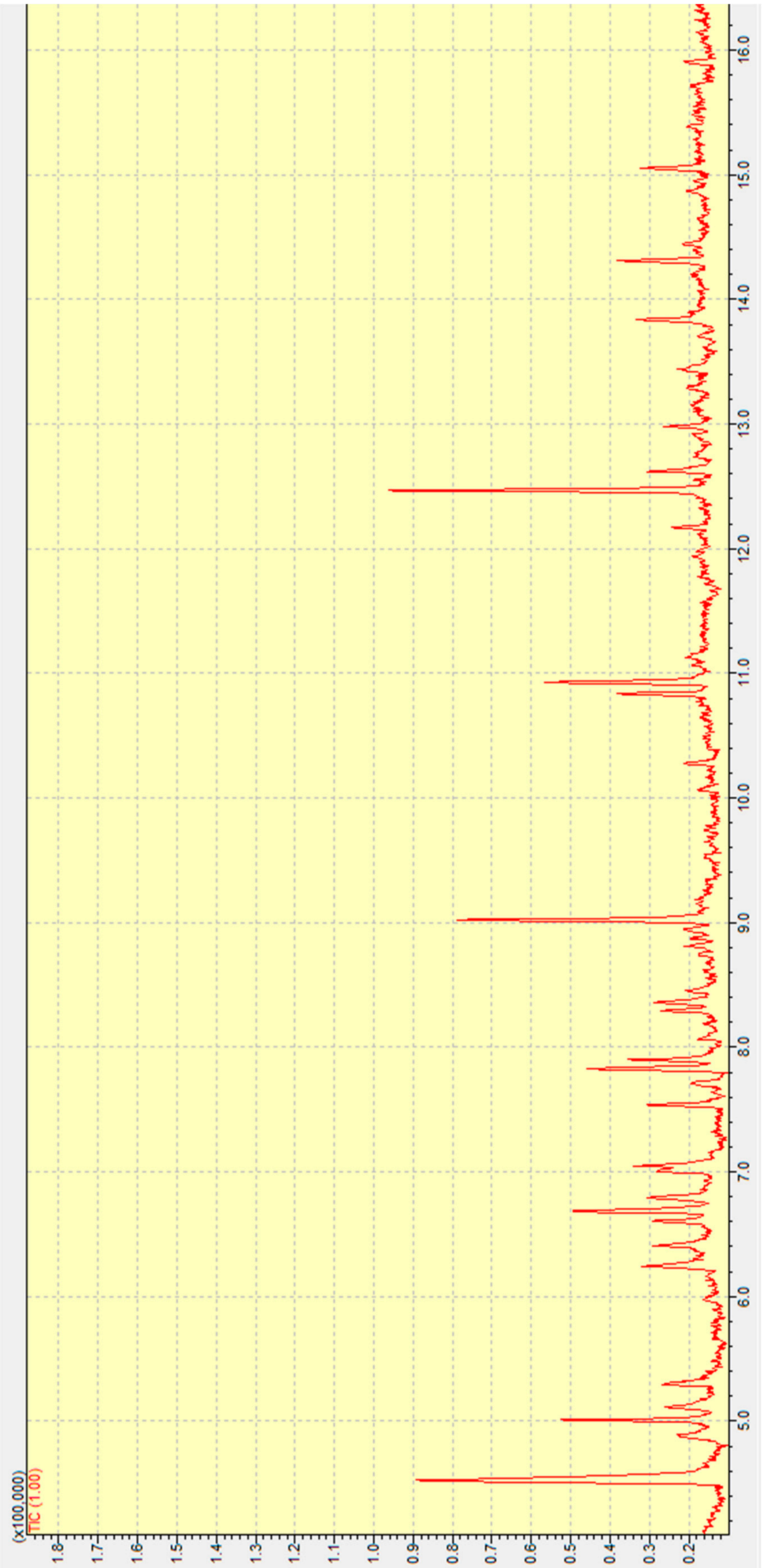

Figure S25. Representative chromatogram of the 1D sample

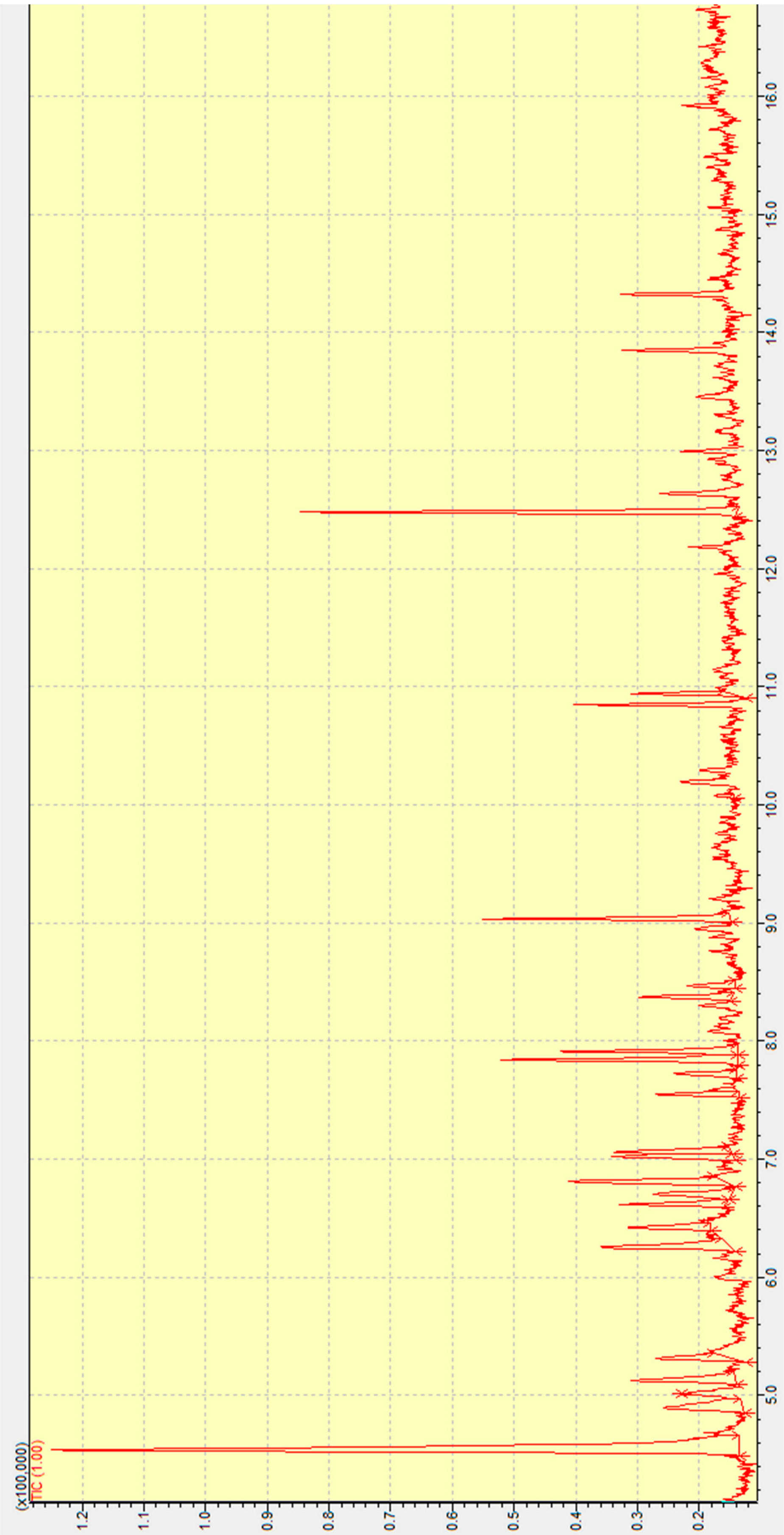

Figure S26. Representative chromatogram of the 2D sample

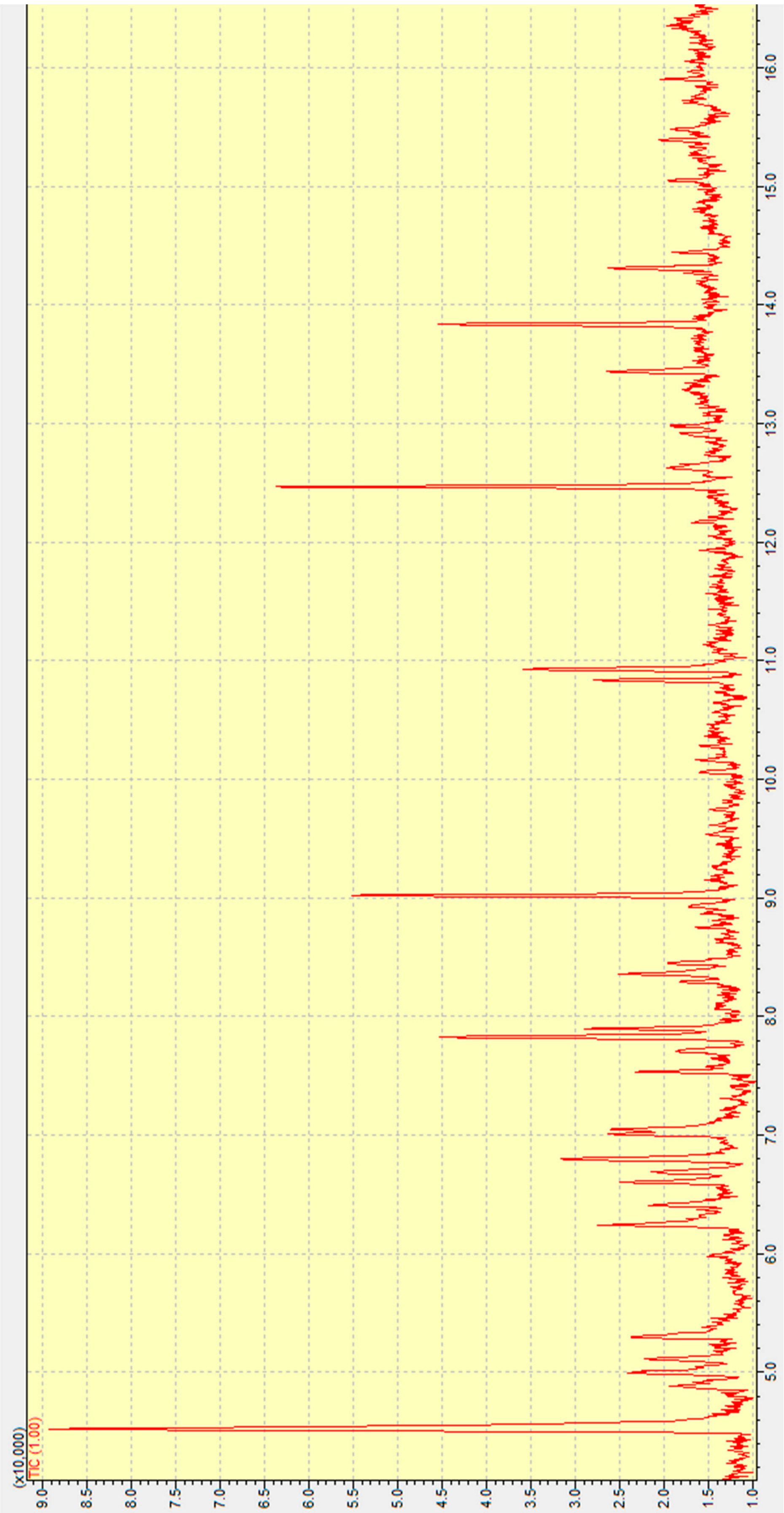

Figure S27. Representative chromatogram of the 3D sample

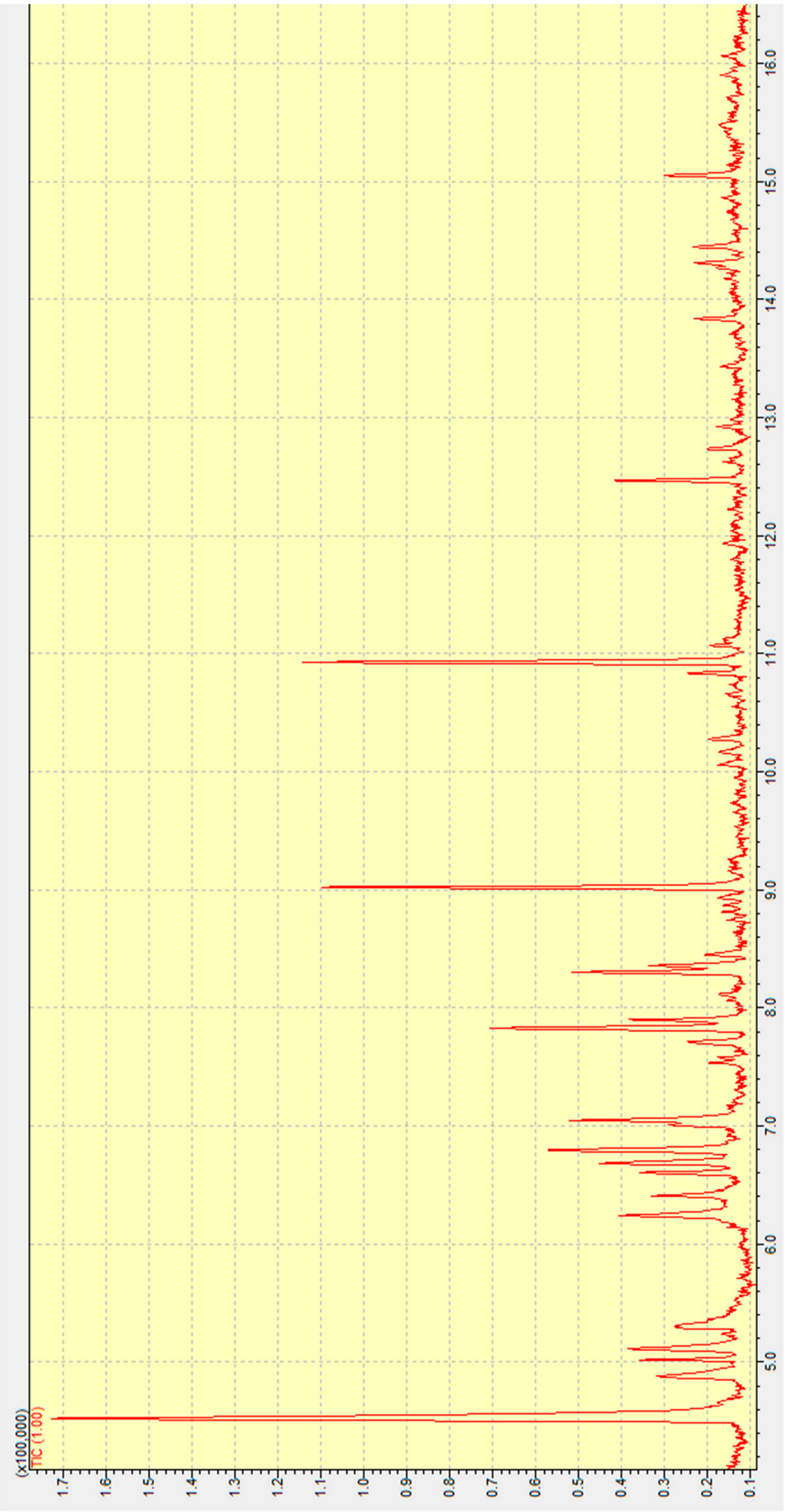

Figure S28. Representative chromatogram of the 4D sample

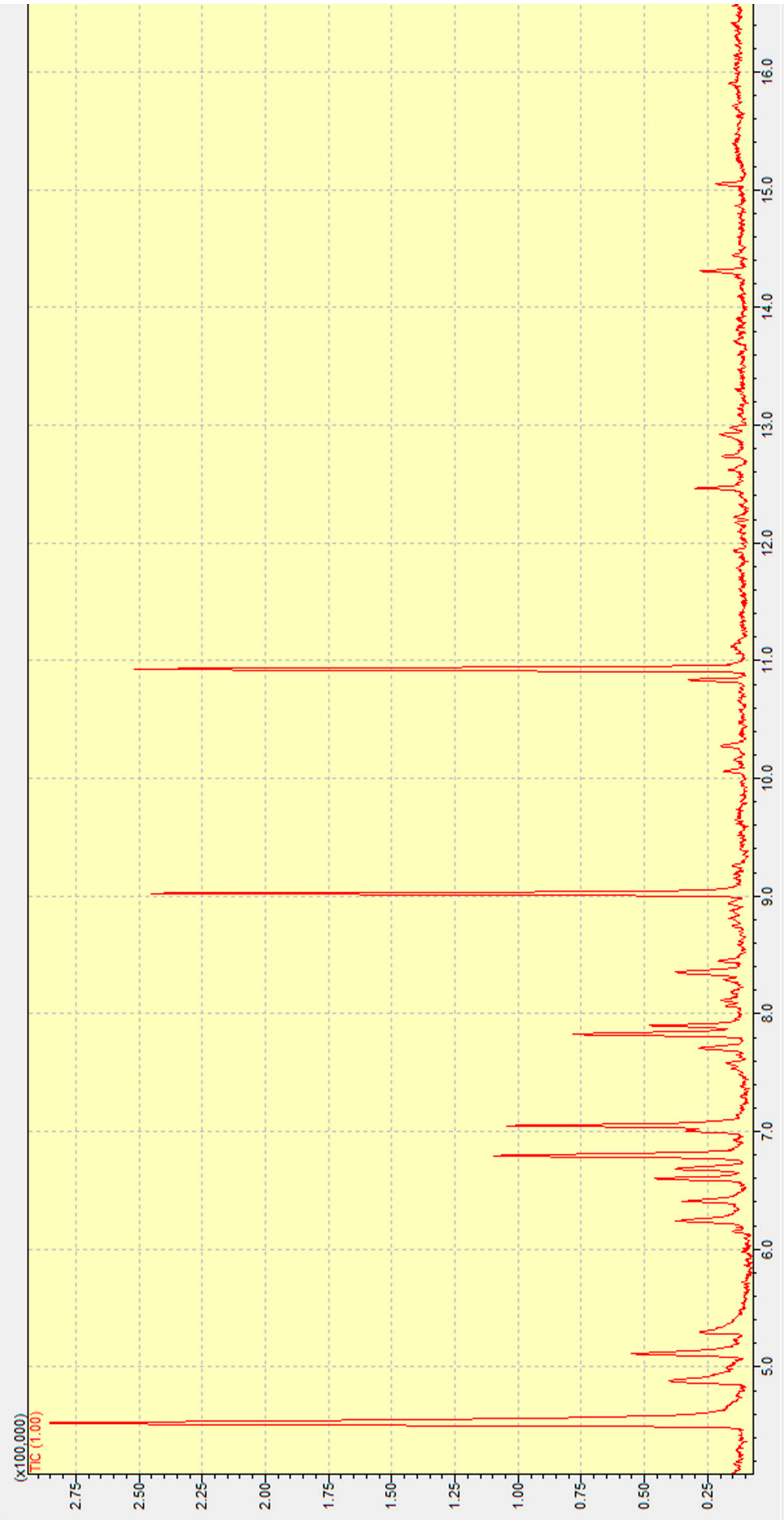

Figure S29. Representative chromatogram of the 5D sample
